# Genetic and epidemiological evidence linking respiratory and musculoskeletal diseases: shared risk factors and intervention windows

**DOI:** 10.1186/s12931-026-03726-y

**Published:** 2026-06-09

**Authors:** Olivia Murrin, Bethany Voller, Ruby M. Woodward, Lucía A. Carrasco-Ribelles, Deniz Türkmen, Qingze Gu, Kate Boddy, Leon Farmer, Mary Mancini, Sara Khalid, Nicholas Aveyard, Chris Fox, Jack Bowden, Frank Dudbridge, Sarah E. Lamb, Jane A. H. Masoli, Concepción Violán, Luke C. Pilling, Timothy M. Frayling, João Delgado

**Affiliations:** 1 Department of Clinical and Biomedical Sciences, Faculty of Health and Life Sciences, University of Exeter, Exeter, UK; 2 Division of Public Health & Epidemiology, School of Medical Sciences, University of Leicester, Leicester, UK; 3 Unitat de Suport a La Recerca Metropolitana Nord, Fundacio Institut Universitari Per a La Recerca a L’Atencio Primaria de Salut Jordi Gol I Gurina (IDIAPJGol), Barcelona, 08007 Spain; 4 Grup de Recerca en Impacte de Les Malalties Cròniques I Les Seves Trajectòries (GRIMTra) (2021 SGR 01537), Institut Universitari d’Investigació en Atenció Primaria Jordi Gol (IDIAPJGol), Mare de Déu de Guadalupe, 2, Barcelona, 08303 Spain; 5 Red de Investigación en Cronicidad, Atención Primaria y Prevención y Promoción de La Salut (RICAPPS), Instituto de Salud Carlos III (ISCIII), Avenida Monforte de Lemos,5, Madrid, 28029 Spain; 6 Nuffield Department of Orthopaedics Rheumatology and Musculoskeletal Sciences, Centre for Statistics in Medicine, University of Oxford, Oxford, UK; 7 Department of Health and Community Sciences, Faculty of Health and Life Sciences, University of Exeter, Exeter, UK; 8 Public and Patient Involvement Representative, Exeter, UK; 9 Royal Devon University Healthcare NHS Foundation Trust, Barrack Road, Exeter, EX2 5DW UK; 10 Novo Nordisk Research Centre, Roosevelt Drive, Headington, Oxford, UK; 11 Leicester British Heart Foundation Centre of Research Excellence, University of Leicester, Leicester, UK; 12 Department of Public Health and Sports Sciences, Faculty of Health and Life Sciences, University of Exeter, Exeter, UK; 13 Germans Trias I Pujol Research Institute (IGTP), Barcalona, 08916 Spain; 14 Department of Medicine, Universitat Autònoma de Barcelona, Plaça Cívica, 1, Barcelona, 08193 Spain; 15 Department of Genetic Medicine and Development, Faculty of Medicine, University of Geneva, 1 Rue Michel-Servet, Geneva 4, CH-1211 Switzerland

**Keywords:** Multimorbidity, Respiratory diseases, Chronic obstructive pulmonary disease (COPD), Asthma, Rheumatoid arthritis, Osteoarthritis, Polymyalgia rheumatica, Mendelian randomisation, Electronic health records, Causal inference

## Abstract

**Background:**

We previously identified genetic correlation between pairs of musculoskeletal (MSK) and respiratory conditions. Strategies to prevent or delay their onset remain underexplored in the context of multimorbidity. This study investigated whether MSK–respiratory disease pairs show evidence of potential causal relationships, identified modifiable risk factors, and quantified intervention windows to prevent progression to multimorbidity.

**Methods:**

We examined combinations of one respiratory condition (asthma, COPD) and one MSK condition [rheumatoid arthritis (RA), osteoarthritis (OA), polymyalgia rheumatica (PMR), psoriasis]. Two-sample Mendelian randomisation (MR) evaluated potential causal relationships in both directions. Linked electronic health records from CPRD (*N =* 11,042,985; age ≥ 40 years) were used to assess longitudinal disease trajectories, prognostic consequences, and mediation by potentially modifiable or treatable factors.

**Results:**

We found evidence for bidirectional relationships between COPD and RA/OA (ORs 1.10–1.19) and between asthma and RA/OA (ORs 1.03–1.14). COPD genetic liability also increased PMR risk (OR 1.14, 95% CI 1.03–1.25). Psoriasis liability increased risk of COPD (OR 1.04, 95% CI 1.02–1.07) and asthma (OR 1.05, 95% CI 1.03–1.08).

Across disease pairs, the median interval between first and second diagnoses was 5–13 years, indicating a substantial window for intervention. Obesity, smoking, hypertension, and thyroid conditions were risk factors common across all studied conditions. Mediation analyses suggested reduced physical activity and lipid changes partially contributed to the onset of respiratory disease following MSK conditions. Colocalisation identified genetic variants causal for specific condition pairs (implicating genes *IFIH1*, *APOE*, and *CXCR5*), highlighting inflammatory and lipid pathways.

**Conclusions:**

MSK and respiratory conditions commonly develop sequentially over many years. Targeted strategies promoting physical function, optimising lipid and cardiovascular risk management may help delay or prevent multimorbidity progression.

**Supplementary Information:**

The online version contains supplementary material available at https://doi.org/10.1186/s12931-026-03726-y.

## Introduction

Musculoskeletal (MSK) conditions are common worldwide: at least 7.6 million people have rheumatoid arthritis (RA) [1] and 595 million people have osteoarthritis (OA) [2]. Respiratory diseases are also widespread with an estimated 262 million people affected by asthma [3] and 174 million by chronic obstructive pulmonary disease (COPD) [4]. COPD is responsible for approximately 3.5 million deaths annually [5].

We previously identified genetic overlap between pairs of MSK and respiratory conditions, indicating potential shared mechanisms (e.g., genetic correlation between RA and COPD is 35%, (95% CI 27–43) [6]. People with RA were also 80%, (95% CI 75–84) more likely to have COPD compared to those without RA after adjusting for age and sex. The co-occurrence of multiple long-term conditions, multimorbidity, is associated with higher mortality rates [7] and reduced quality of life [8]. Some combinations of respiratory and MSK conditions could lead to worsening prognosis. Understanding the causality of the condition relationships and clarifying these prognostic differences could help prioritise cases for screening and intervention.

Multimorbidity studies have largely examined broad patterns of disease without informing specific interventions [9]. In turn, studies that do investigate risk factors either focus on single disease impact [10] or multimorbidity counts that are agnostic to specific combinations of diseases [11]. Disease onset can lead to barriers to maintaining a healthy lifestyle, for example, pain in OA [12] and RA [13] and breathlessness in COPD [14] can reduce the ability to exercise. Reviews on interventions for multimorbidity call for targeting patient-centred outcomes such as physical activity (PA) and disease-specific interventions relevant to multimorbidity [15]. Distinct clinical windows for intervention opportunities may exist: 1) before first disease onset, 2) between onset of a first and a second disease, and 3) after multimorbidity is established, each potentially requiring unique intervention strategies. Here, we focus on identifying potential interventions to prevent, delay or treat multiple long-term conditions. We use Mendelian randomization to identify which genetically linked disease pairs and risk factors are most likely to be causal, and therefore promising targets for intervention and mediation analyses in observational data.

To explore intervention opportunities, we prespecified the following causal questions: (1) Do causal relationships exist between musculoskeletal (MSK) and respiratory conditions? (2) Are there identifiable windows for intervention? (3) Which combinations of conditions lead to the worst prognosis? (4) Which modifiable risk factors can be targeted to prevent future disease, including those that may mediate the onset of a second condition after the first? and (5) Are there shared biological pathways that could inform drug repurposing for multimorbidity? We used two-sample Mendelian randomization (MR), which under specific assumptions estimates causal effects of genetically proxied exposures on outcomes, to test causal relationships between diseases and risk factors, and population-representative electronic health record data to examine temporal relationships and prognosis.

## Methods

### Data sources

We investigated pairs of diseases that included respiratory and MSK associated conditions that we have previously found to be genetically correlated (Supporting information Table S1). Respiratory conditions included COPD and asthma, and MSK associated conditions include polymyalgia rheumatica (PMR), OA, RA, and psoriasis, which can be associated with psoriatic arthritis. Code lists for diseases are available at Murrin et al. [1].

Genetic associations with disease outcomes were identified from our meta-analyses of 72 long-term conditions in the GEMINI research collaborative using two large cohort studies: UK Biobank (*N =* 502,000) [16], FinnGen (release 9; *N =* 377,000) [17] and condition-specific consortium data when available [6]. These summary statistics are available from (https://doi.org/10.5281/zenodo.14284046).

We also used summary statistics of recently published GWAS from leading consortia for ten potential risk factors: BMI [18], waist-hip ratio [19], cholesterol (LDL, HDL, triglycerides) [20], educational attainment [21], smoking status (ever smoked, and separately smoking intensity in smokers) [22], alcohol consumption [22], and PA [23].

Observational data were analysed from the UK Clinical Practice Research Datalink Aurum (CPRD) [24]. We replicated the observed time-to-event analyses (described below) in the Spanish Information System for Research in Primary Care (SIDIAP) [25]. Data included *N =* 11,046,182 CPRD (2010–2020) and *N =* 2,625,989 SIDIAP (2013–2020) registered patients over 40 years of age. Clinical records in CPRD codes are a mixture of Read v2 Codes, EMIS and SNOMED while in SIDIAP they are recorded using the International Classification of Disease, version 10 (ICD-10) (Supplementary Information -Observational Note).

We ascertained 26 variables to be tested as potential covariates in observational pairwise analyses. These variables include diseases, lifestyle and demographic variables, health biometric data and biomarkers. Potentially relevant disease comorbidities were ascertained using the GEMINI code lists (Supporting information Table S2). Deprivation was measured using CPRD-linked data (Townsend Index of Multiple Deprivation 2019) [26]. Biometric and biomarker covariates were ascertained using code lists and data cleaning protocols adapted from the “EHRBiomarkr” package (https://github.com/Exeter-Diabetes/EHRBiomarkr) [27]. Biometric variables such as blood pressure are frequently missing not-at-random in routine electronic health records [28]. Continuous variables were categorised according to clinically relevant cut-offs (S2) and analysed as nominal categorical variables, with missing values retained as an explicit category (“unknown”) to preserve sample size and reflect potential informativeness of missingness.

### Ethics

This study was approved by the relevant ethics committees:

UK Biobank: North West MREC (11/NW/0382), Application 14,631. CPRD ISAC: 23_003109. SIDIAP Scientific and Ethical Committees: 19/518-P (18/12/2019). All analyses used anonymised data.

### Mendelian randomisation

We used two-sample Mendelian randomisation (MR) methods to estimate the bidirectional causal effect between each respiratory and MSK condition, and to estimate the causal effect of potential risk factors on respiratory and MSK conditions (S2). Mendelian randomization (MR) analysis can estimate the causal effect between an exposure and an outcome by using genetic variants as instrumental variables, thereby minimizing bias from confounding and reverse causation present in conventional observational studies. Selected genetic instruments were 1) strongly associated with the exposure (*p <* 5 × 10⁻⁸), 2) common (minor allele frequency [MAF] > 0.1%), and 3) independent (r^2^ < 0.01). Due to GWAS data availability analyses included participants genetically similar to the 1000 Genomes project European (EUR) subset [29].

Analyses were performed using the ‘TwoSampleMR’ R package v0.6.15 [30]. Multiple sensitivity analyses were performed after a primary analysis using inverse variance weighted (IVW) regression, including the weighted median and weighted mode estimators, and MR-Egger. We assessed instrument heterogeneity using Cochran’s *Q* statistic and applied Radial MR to identify pleiotropic outliers [31, 32] and Steiger filtering to remove variants with greater effect on the outcome than on the exposure [33]. Detailed MR rationale is available from Voller et al. [34].

A second MR analysis excluding the human leukocyte antigen (HLA) region was performed to reduce the influence of highly pleiotropic variants, considering that many pairs of conditions included one or both with autoimmune components. MR results for disease relationships are reported as odds ratios, denoting the change in risk of the outcome based on a doubling of genetic liability to the exposure disease [35]. Multivariable MR (MVMR), including a Q-minimisation estimate, which is more robust against conditionally weak instruments that can arise from sample overlap [36], assessed whether the effects of genetic liability to each disease were robust when accounting for potentially correlated risk factors [37] (see supplemental material-MR Method overview).

This study complied with the STROBE-MR guidelines for reporting MR [38].

### Observational analyses

#### Observational time to event models

We estimated the time to a second condition after first LTC. We applied robust methods for controlling confounding, including matching and weighting exposure groups on important confounders, while acknowledging that residual bias may remain [39].

Longitudinal analyses assessed the effect of disease A on the subsequent incidence of disease B. For everyone with disease A (the exposed group), the index date was defined as the earliest date of (1) reaching age 40 years, (2) GP registration date, or (3) the date of diagnosis with disease A.

A control group free from both diseases at the index date was matched 1:1 on age and sex (Supporting Information Tables S3–S4).

Propensity scores (*p*) were estimated using logistic regression models including covariates significantly associated with outcome disease B [40] (chosen covariates listed in supplementary information). Overlapping weights [41] derived from these scores were applied to balance differences in baseline characteristics between exposed and control groups:

$$w_i=(1-p_i)+(1-A_i)p_i,\;\mathrm{Where}\;A_{\mathit i}=1\;(has\mathit\;disease\mathit\;A)$$

We used overlap-weighted Cox proportional hazards models to estimate the association minimally confounded by measured covariates between prior disease A and incident disease B in the overlap population.

E-values were calculated for observational estimates. The E-value for the point estimate reflects the minimum strength of association an unmeasured confounder would need with both exposure and outcome to fully explain the observed association, whereas the E-value for the confidence interval reflects the strength required to shift the estimate to include the null (listed in brackets in the tables) [42].

#### Prognostic consequences

We compared mortality rates between those with disease A, disease B, and both (A&B), using people without either disease as the reference group. The index date for exposed individuals was the earliest of reaching age 40 years, CPRD registration, or diagnosis with the target condition(s), with the date of the second diagnosis used for those with both conditions (A&B group). The multimorbid group was matched 1:1 to single disease groups on age, sex, and calendar year of index date. A single control individual without either disease was then matched on age and sex and shared across the three exposed groups. This strategy allows us to directly compare hazard ratios (HRs) between multimorbid and single condition groups [43]. Each control was assigned the index date of their matched multimorbid case (Supporting Information Table S5). During the analyses we separated the A&B group by order of disease diagnosis (i.e., A before B and B before A).

Mortality was defined as the earliest date from the Office for National Statistics (ONS) death registration and CPRD AURUM data using an algorithm prioritising ONS death dates with a higher match rank [44]. Cox proportional hazards models estimated the risk of 10-year incident all-cause mortality. Covariates were selected using LASSO-penalised Cox regression (cv.glmnet) with tenfold cross-validation. The optimal penalty parameter (λ) was chosen as lambda.min, the value minimising the mean cross-validated partial likelihood deviance. Variables with non-zero coefficients at this λ were retained from 26 candidate covariates (S2) [45].

The number of hospitalisations (1 year of follow-up) for all admissions and unplanned admissions were extracted from the CPRD-linked hospital episode statistics admitted patient care. Negative binomial models compared counts across groups. These models were adjusted for relevant covariates identified using ‘glmnet’ LASSO regression variable selection [45].

#### Disease sequences and the window for intervention

We examined temporal trajectories to characterise the diagnostic sequence between respiratory and MSK conditions. For each disease pair, we estimated the proportion of individuals diagnosed with A then B and vice-versa, the mean age at diagnosis, and the mean and median times between diagnoses. The mean and median time between conditions were considered as a potential window for intervention. The standard deviation (SD) and interquartile ranges (IQR) were reported.

#### Risk factor prioritisation

We prioritised risk factors that were precipitated by or exacerbated by the first condition and that could be targeted within the intervention window**.** A time-to-event mediation analysis estimated the proportion of the observed relationship (e.g., A leading to B) that is explained by a risk factor. Candidate mediators were selected based on: 1) MR evidence that condition A influences the mediator (Supporting information Tables S6), and 2) a Z test comparing the adjusted and unadjusted observed odds ratio of the disease pair relationship (Supporting information Tables S7) [46]. This time-to-event mediation analysis is an extension of the pair time to event analyses in CPRD described above and shown in Table 1 [41, 47]. The candidate mediator is excluded from the propensity score. Mediator values were defined using the first recorded measurement after the index date; individuals with missing mediator data were excluded.

**Table 1:** Causal and observed relationships: Mendelian randomisation (MR) results are expressed on the exp(b × log₂) scale, representing the risk associated with a doubling of genetic liability to the exposure disease. Observational estimates indicate risk of disease onset within 10 years of follow-up. MR results are shown with and without HLA (chr6:25–34 Mb); attenuation after HLA exclusion suggests auto-immune related pleiotropy and causal estimates should be treated with caution. E-value indicates the minimum confounder strength required to explain away the observed effect; E-value CI (bracketed) is calculated for the confidence interval bound closest to the null

		**Mendelian randomization**		**Observational estimates**			
**Disease pair**	**Direction**	**IVW MR (OR)**	**IVW MR no HLA (OR)**	**CPRD (HR)**	**CPRD E-value**	**SIDIAP (HR)**	**SIDIAP E-value**
asthma & OA	asthma → OA^‡^	1.03 (1.02 to 1.05)***	1.03 (1.01 to 1.05)***	1.36 (1.34 to 1.37)***	2.05 (2.02)	1.21 (1.18 to 1.25)***	1.72 (1.63)
	OA → asthma	1.14 (1.09 to 1.19)***	1.14 (1.08 to 1.19)***	1.54 (1.49 to 1.58)***	2.45 (2.35)	1.29 (1.25 to 1.33)***	1.90 (1.80)
asthma & PMR	asthma → PMR	1.01 (0.95 to 1.08)	1.03 (0.96 to 1.10)	1.38 (1.33 to 1.43)***	2.10 (2.00)	1.21 (1.10 to 1.33)***	1.71 (1.44)
	PMR → asthma	1.02 (0.98 to 1.07)	^$^	1.36 (1.26 to 1.47)***	2.06 (1.83)	1.05 (0.87 to 1.27)	1.29 (1.00)
asthma & psoriasis	asthma → psoriasis	1.05 (1.01 to 1.10)*	1.03 (0.99 to 1.08)	1.27 (1.24 to 1.31)***	1.86 (1.78)	1.11 (1.02 to 1.20)*	1.45 (1.17)
	psoriasis → asthma	1.02 (1.00 to 1.04)*	1.05 (1.03 to 1.08)***	1.27 (1.23 to 1.32)***	1.86 (1.76)	1.12 (1.02 to 1.22)*	1.47 (1.16)
asthma & RA	asthma → RA	1.19 (1.13 to 1.24)***	1.12 (1.07 to 1.18)***	1.48 (1.43 to 1.54)***	2.33 (2.21)	1.25 (1.08 to 1.45)**	1.80 (1.36)
	RA → asthma	1.07 (1.05 to 1.10)***	1.07 (1.04 to 1.10)***	1.40 (1.31 to 1.49)***	2.14 (1.94)	1.35 (1.18 to 1.55)***	2.04 (1.64)
COPD & OA	COPD → OA	1.10 (1.06 to 1.13)***	1.10 (1.06 to 1.13)***	1.11 (1.08 to 1.15)***	1.47 (1.38)	1.05 (1.02 to 1.08)**	1.28 (1.15)
	OA → COPD	1.18 (1.13 to 1.24)***	1.19 (1.13 to 1.25)***	1.19 (1.16 to 1.21)***	1.66 (1.60)	1.05 (1.03 to 1.08)***	1.29 (1.19)
COPD & PMR	COPD → PMR	1.14 (1.03 to 1.25)**	1.14 (1.03 to 1.25)**	1.10 (1.02 to 1.19)*	1.44 (1.15)	1.03 (0.94 to 1.13)	1.21 (1.00)
	PMR → COPD	1.05 (1.02 to 1.08)**	^$^	1.20 (1.14 to 1.26)***	1.69 (1.55)	0.96 (0.84 to 1.11)	1.24 (1.00)
COPD & psoriasis	COPD → psoriasis	1.08 (1.01 to 1.15)*	1.08 (1.01 to 1.15)*	1.45 (1.34 to 1.57)***	2.26 (2.01)	1.26 (1.16 to 1.37)***	1.83 (1.59)
	psoriasis → COPD	1.01 (0.99 to 1.03)	1.04 (1.01 to 1.07)**	1.37 (1.33 to 1.41)***	2.07 (1.99)	1.10 (1.04 to 1.17)**	1.43 (1.23)
COPD & RA	COPD → RA^‡^^†^	1.20 (1.12 to 1.28)***	1.19 (1.11 to 1.27)***	1.87 (1.75 to 2.00)***	3.15 (2.90)	1.40 (1.20 to 1.64)***	2.15 (1.68)
	RA → COPD	1.06 (1.04 to 1.09)***	1.10 (1.07 to 1.12)***	1.68 (1.60 to 1.76)***	2.74 (2.58)	1.45 (1.31 to 1.60)***	2.25 (1.95)

^*^p < 0.05, ^**^p < 0.01, ^***^p < 0.001

^$^Analysis excluding HLA region not possible due to insufficient genetic instruments

^‡^Partial MVMR Attenuation after adjustment for IL6

^†^Partial MVMR Attenuation after adjustment for asthma

This approach separates the total effect of Disease A on Disease B into indirect and direct effects while accounting for censored time-to-event data [48]. The model reports the proportion of the effect explained by the mediator. *P* values were corrected for multiple testing using the Benjamini–Hochberg method for each disease pair [49]. Mediation analyses were performed using the R “mediation” package [50]. We do not report results where the direct and indirect effects have opposite directions as there is no clinically valid interpretation of the findings.

This study complied with the RECORD (Reporting of Studies Conducted using Observational routinely collected Data) statement [51].

### Identifying shared genetic mechanisms

#### Colocalisation

For each combination of respiratory and MSK conditions, we identified the genome-wide significant variants (*p <* 5 × 10⁻⁸) shared by the disease pair. Starting with the variant with the smallest p-value, we defined regions of ± 250 kb around each variant, until all variants were assigned to a region. To investigate whether the disease pairs shared causal variants, we then performed statistical colocalisation using the ‘coloc.abf*’* function from the ‘coloc’ R package (version 5.2.3) [52].

Coloc is a Bayesian method that computes posterior probabilities(PP) for five hypotheses (H0–H4 defined in Supplementary Information) per genomic region; we focus on PPH4 (a single shared causal variant). For regions with strong evidence of a shared causal variant, coloc can identify possible variants and estimate the probabilities of each being the specific shared variant (referred to as SNP PPH4). We used the default prior probabilities: ${p}_{1}=1\times {10}^{-4}, {p}_{2}=1\times {10}^{-4}, {p}_{12}=1\times {10}^{-5}$, where ${p}_{1}$ and ${p}_{2}$ are the probabilities that a random SNP in the region is causally associated with trait 1 or trait 2 respectively, and ${p}_{12}$ is the probability that a variant is causal for both traits. We considered evidence for colocalisation sufficient if the combined posterior probability of H3 and H4 (PPH3 and PPH4, respectively) was high: PPH3 + PPH4 ≥ 0.8 suggests an association with both traits, and where PPH4/PPH3 > 5 this suggests sufficient support to consider the colocalising signal ‘convincing’. These methods are described in further detail in Voller et al. [34]. For each region with evidence of colocalisation, we extracted the posterior probabilities for each SNP in the region to be causal conditional on H4 being true.

### Follow-up of shared signals

To assess whether circulating proteins play a role in the shared genetic architecture between disease pairs, we used proteomic data for 2,923 plasma proteins produced by the Olink platform in the UK Biobank pharma proteomic project (*N =* 48,195 European individuals) to identify whether colocalising variants were protein quantitative trait loci (pQTLs) (*p <* 5 × 10^–5^) [53]. Variants within 1 MB of the encoded gene’s transcription start site were classified as *cis-*pQTLs, all others as *trans*-pQTLs.

Where colocalisation supported a causal role of a protein in a trait-pair, we assessed the druggability of the protein (the likelihood that a protein can be targeted by an existing or developable drug), using DrugBank [54], and the Drug-Gene Interaction Database [55]. We highlighted cases where druggable proteins were targeted by existing or investigational therapies.

## Results

We analysed data from individuals aged ≥ 40 years in CPRD (*N =* 11,042,985). At study entry, mean age was 55.9 years (SD 14.9) and 50.2% were women; deprivation was broadly distributed (IMD1 21.4%, IMD5 18.1%). We built age and sex matched cohorts for our time-to-event analyses.-In the time-to-event analyses, the mean age at index ranged from 52.6 years (asthma, excluding prior OA) to 77.3 years (PMR, excluding prior asthma) (S3–S4). The female proportion ranged from 23% (COPD, excluding prior OA) to 76% (RA, excluding prior COPD).

For participants in the prognostic consequences analysis, the mean age at index ranged from 55.3 years (asthma then psoriasis) to 78.1 years (PMR then COPD) (S5). Patients with asthma or COPD who later developed an MSK condition were more likely to be in the most deprived IMD quintile (IMD5)—up to 28.5%—whereas MSK patients without a lung condition generally showed similar deprivation to their matched controls; the lowest IMD5 proportion was among those with PMR but without COPD (9.2%).

**Fig. 1:**
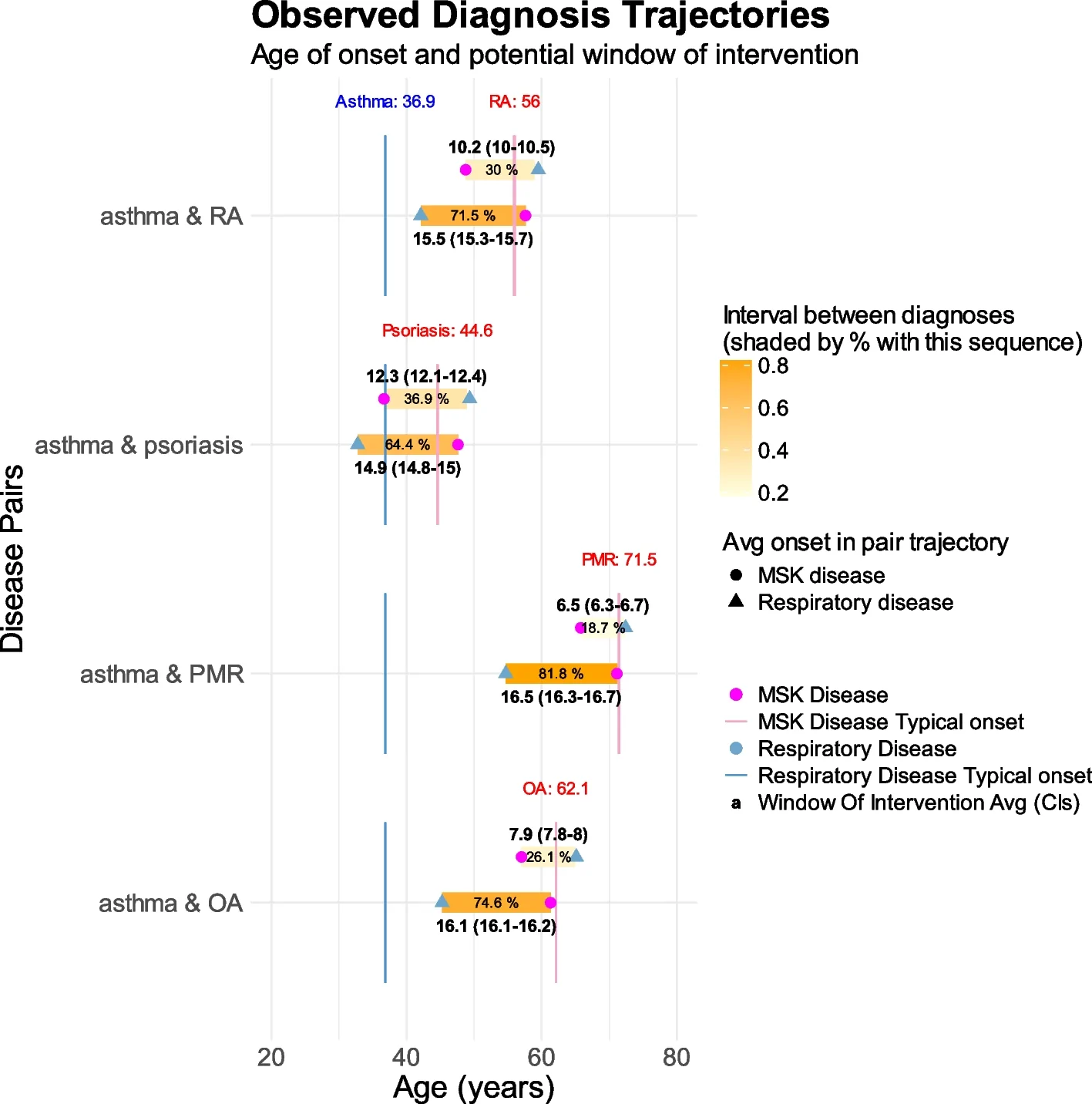
Observed asthma and MSK disease trajectories. Vertical lines show the overall mean age at onset in CPRD for each disease (i.e., across all patients with that disease, not limited to multimorbid pairs). Circles (MSK) and triangles (respiratory) mark the mean age at onset within the specific disease-pair trajectory (i.e., among patients who developed disease A then disease B). The horizontal segment spans the mean interval between the two diagnoses, with the number above or below the segment giving that mean interval. For visual clarity we plot means with confidence intervals; median ages and IQRs are reported in the main text and Supplementary Table S9

**Fig. 2:**
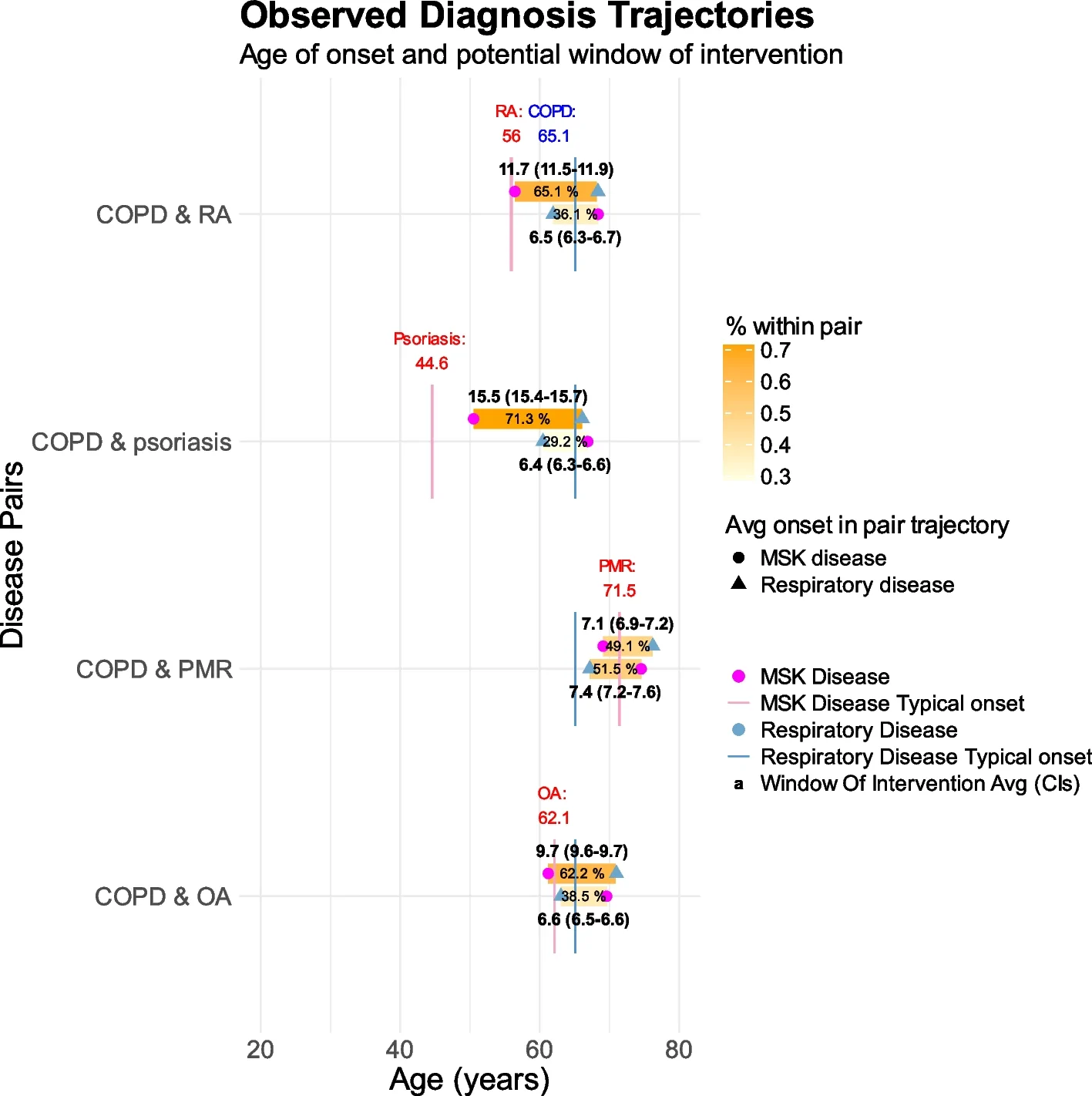
Observed COPD and MSK disease trajectories. Vertical lines show the overall mean age at onset in CPRD for each disease (i.e., across all patients with that disease, not limited to multimorbid pairs). Circles (MSK) and triangles (respiratory) mark the mean age at onset within the specific disease-pair trajectory (i.e., among patients who developed disease A then disease B). The horizontal segment spans the mean interval between the two diagnoses, with the number above or below the segment giving that mean interval. For visual clarity we plot means with confidence intervals; median ages and IQRs are reported in the main text and Supplementary Table S9

For each long-term condition (LTC) pair—stratified by order of onset and by respiratory index disease e.g. asthma preceding MSK conditions—we report: (1) causal estimates from two-sample MR (Table 1; Supplementary Material – Comprehensive MR results, S8 Table), expressed as odds ratios (OR) per doubling of genetic liability (OR = exp(β × log 2)) The observed time to event estimates are reported in Table 2; (2) disease trajectories and time between diagnoses derived from longitudinal EHRs (Figs. 1 and 2; S12 Table).

**Table 2:** Consequences of multimorbidity pairs: mortality estimates reflect 10-year mortality risk. Incidence rate ratios (IRRs) compare hospitalisation rates per person-year between groups during the first year of follow-up. All hazard ratio (HR) and IRR estimates are relative to individuals with neither condition. E-value indicates the minimum confounder strength required to explain away the observed effect; E-value CI (bracketed) is calculated for the confidence interval bound closest to the null. *p* < 0.05; * *p* <0.01; ** *p* < 0.001

		**Mortality**		**Hospitalisations**			
**Disease pair**	**Variable**	**Mortality CPRD (HR)**	**Mortality E-value**	**All hospitalisations (IRR)**	**All admissions E-value**	**Unplanned hospitalisations (IRR)**	**Unplanned admissions E-value**
Asthma & OA	Only OA	0.95 (0.94 to 0.97)***	1.27 (1.21)	1.27 (1.25 to 1.29)***	1.86 (1.82)	1.13 (1.10 to 1.15)***	1.50 (1.44)
	Only asthma	1.38 (1.36 to 1.40)***	2.11 (2.07)	1.25 (1.24 to 1.27)***	1.82 (1.78)	1.49 (1.47 to 1.52)***	2.35 (2.29)
	asthma before OA	1.12 (1.10 to 1.14)***	1.49 (1.44)	1.51 (1.49 to 1.54)***	2.39 (2.34)	1.55 (1.52 to 1.58)***	2.47 (2.41)
	OA before asthma	1.11 (1.09 to 1.14)***	1.47 (1.41)	1.45 (1.42 to 1.48)***	2.25 (2.19)	1.55 (1.51 to 1.59)	2.47 (2.38)
Asthma & PMR	Only PMR	1.09 (1.04 to 1.14)***	1.40 (1.26)	1.23 (1.18 to 1.28)***	1.76 (1.63)	1.26 (1.19 to 1.33)***	1.83 (1.67)
	Only asthma	1.31 (1.26 to 1.36)***	1.95 (1.83)	1.21 (1.16 to 1.26)***	1.71 (1.59)	1.45 (1.38 to 1.53)***	2.26 (2.10)
	asthma before PMR	1.33 (1.27 to 1.39)***	1.99 (1.86)	1.42 (1.36 to 1.48)***	2.18 (2.05)	1.73 (1.64 to 1.83)***	2.86 (2.66)
	PMR before asthma	1.36 (1.28 to 1.45)***	2.06 (1.87)	1.25 (1.16 to 1.34)***	1.80 (1.59)	1.61 (1.48 to 1.76)***	2.60 (2.32)
Asthma & RA	Only RA	1.61 (1.53 to 1.70)***	2.60 (2.43)	1.64 (1.56 to 1.71)***	2.66 (2.50)	1.53 (1.44 to 1.63)***	2.44 (2.23)
	Only asthma	1.30 (1.23 to 1.37)***	1.92 (1.77)	1.24 (1.18 to 1.30)***	1.78 (1.65)	1.53 (1.43 to 1.62)***	2.42 (2.22)
	asthma before RA	1.87 (1.77 to 1.98)***	3.15 (2.93)	1.97 (1.87 to 2.07)***	3.35 (3.16)	2.14 (2.00 to 2.29)	3.71 (3.42)
	RA before asthma	1.95 (1.82 to 2.08)***	3.30 (3.05)	2.09 (1.97 to 2.22)***	3.60 (3.35)	2.22 (2.05 to 2.41)***	3.87 (3.52)
Asthma & Psoriasis	Only asthma	1.29 (1.24 to 1.34)***	1.89 (1.78)	1.28 (1.24 to 1.32)***	1.88 (1.79)	1.54 (1.47 to 1.61)***	2.44 (2.30)
	Only psoriasis	1.17 (1.12 to 1.22)***	1.61 (1.50)	1.12 (1.09 to 1.16)***	1.50 (1.40)	1.12 (1.07 to 1.17)***	1.49 (1.35)
	asthma before psoriasis	1.40 (1.34 to 1.47)***	2.16 (2.02)	1.42 (1.37 to 1.47)***	2.19 (2.08)	1.70 (1.62 to 1.79)***	2.79 (2.62)
	psoriasis before asthma	1.38 (1.32 to 1.45)***	2.11 (1.96)	1.37 (1.32 to 1.43)***	2.08 (1.96)	1.63 (1.54 to 1.72)***	2.65 (2.46)
COPD & OA	Only COPD	2.22 (2.18 to 2.25)***	3.86 (3.79)	1.47 (1.44 to 1.49)***	2.30 (2.24)	1.92 (1.88 to 1.96)***	3.26 (3.17)
	Only OA	1.00 (0.98 to 1.01)	1.07 (1.00)	1.20 (1.19 to 1.22)***	1.70 (1.65)	1.11 (1.09 to 1.14)***	1.47 (1.41)
	COPD before OA	1.79 (1.75 to 1.82)***	2.98 (2.90)	1.66 (1.63 to 1.70)***	2.71 (2.64)	1.99 (1.93 to 2.04)***	3.38 (3.28)
	OA before COPD	1.79 (1.76 to 1.82)***	2.97 (2.91)	1.63 (1.60 to 1.66)***	2.64 (2.58)	1.93 (1.89 to 1.98)***	3.28 (3.19)
COPD & PMR	Only COPD	2.03 (1.94 to 2.13)***	3.48 (3.29)	1.31 (1.25 to 1.38)***	1.95 (1.80)	1.84 (1.73 to 1.95) ***	3.07 (2.85)
	Only PMR	1.08 (1.03 to 1.13)***	1.37 (1.20)	1.11 (1.06 to 1.17)***	1.46 (1.30)	1.16 (1.09 to 1.24) ***	1.59 (1.40)
	COPD before PMR	2.08 (1.97 to 2.20)***	3.59 (3.36)	1.55 (1.46 to 1.64)***	2.46 (2.27)	2.37(1.21 to 1.53) ***	4.17 (3.85)
	PMR before COPD	1.95 (1.85 to 2.06)***	3.31 (3.10)	1.56 (1.47 to 1.66)***	2.50 (2.30)	2.11 (1.97 to 2.27)***	3.64 (3.33)
COPD & RA	Only COPD	2.22 (2.11 to 2.33)***	3.86 (3.63)	1.43 (1.36 to 1.51)***	2.22 (2.07)	1.95 (1.82 to 2.08)***	3.30 (3.04)
	Only RA	1.55 (1.47 to 1.64)***	2.48 (2.31)	1.38 (1.32 to 1.46)***	2.11 (1.96)	1.43 (1.34 to 1.53)***	2.22 (2.01)
	COPD before RA	2.70 (2.54 to 2.88)***	4.85 (4.51)	1.87 (1.74 to 2.00)***	3.14 (2.88)	2.61 (2.40 to 2.83)***	4.65 (4.23)
	RA before COPD	2.91 (2.75 to 3.07)***	5.26 (4.95)	1.95 (1.84 to 2.06)***	3.31 (3.09)	2.54 (2.37 to 2.73)***	4.52 (4.16)
COPD & psoriasis	Only COPD	2.22 (2.14 to 2.31)***	3.87 (3.70)	1.57 (1.51 to 1.63)***	2.52 (2.39)	2.06 (1.96 to 2.16)***	3.53 (3.32)
	Only psoriasis	1.19 (1.15 to 1.24)***	1.67 (1.55)	1.08 (1.04 to 1.13)***	1.39 (1.26)	1.12 (1.07 to 1.18)***	1.50 (1.34)
	COPD before psoriasis	2.40 (2.28 to 2.52)***	4.23 (3.99)	1.54 (1.46 to 1.63)***	2.46 (2.28)	2.19 (2.05 to 2.34)***	3.81 (3.52)
	psoriasis before COPD	2.21 (2.12 to 2.30)***	3.84 (3.66)	1.52 (1.45 to 1.58)***	2.40 (2.27)	2.04 (1.93 to 2.15)***	3.49 (3.27)

**Fig. 3:**
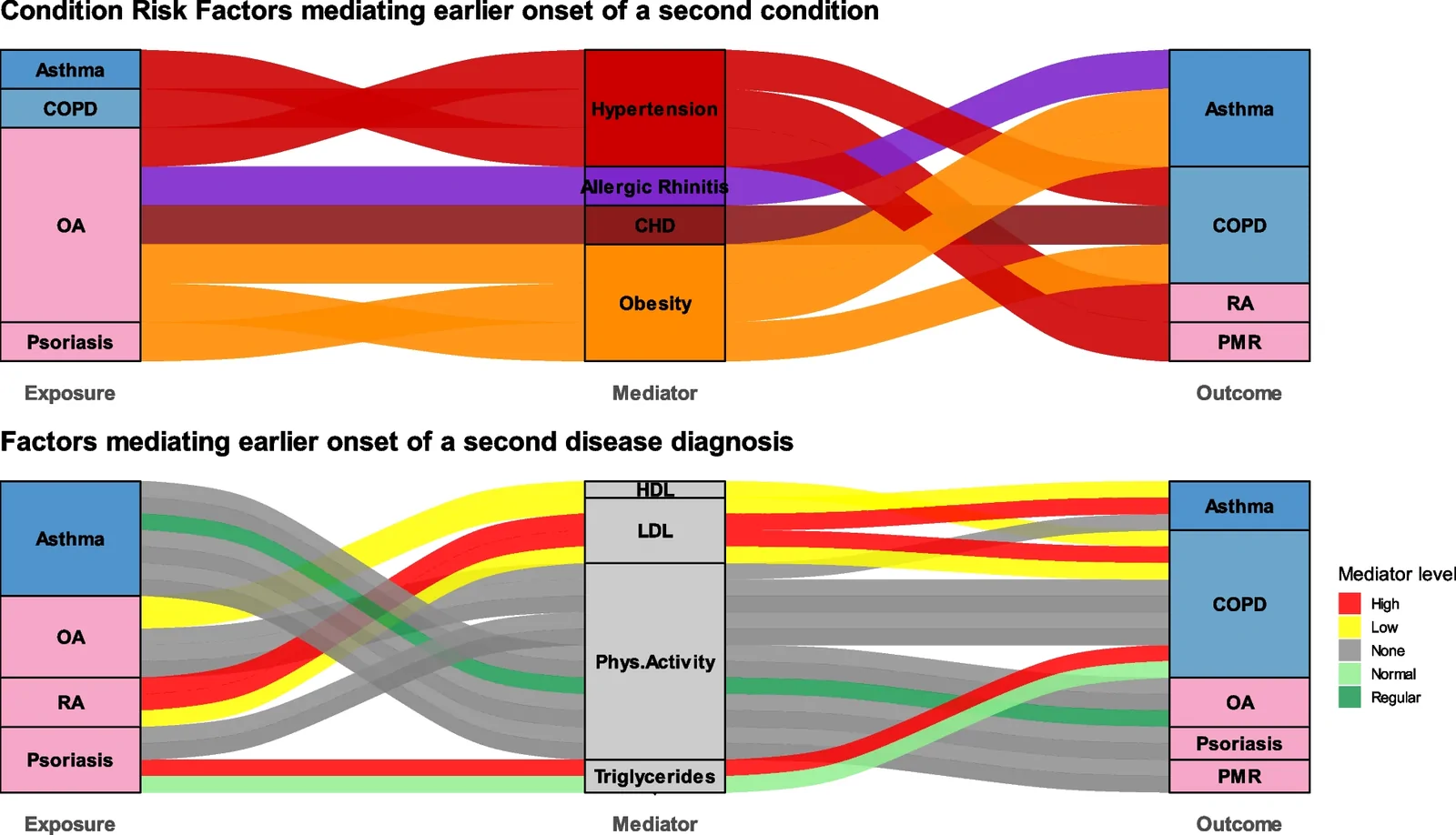
Summary of mediators that accelerate the onset of a subsequent condition. A single line denotes a statistically significant (*p <* 0.05) proportion mediated after correcting for multiple testing (percentage mediated is reported below and in S11

We then report (3) the prognostic consequences of multimorbidity (Table 2); (4) the risk factors implicated by MR for individual diseases (Table 3; S13 Table) and factors that become more relevant after a first condition and may mediate onset of a second condition (Fig. 3; S14 Table). Finally, we report (5) shared genetic architecture across disease pairs from colocalisation and proteomic analyses (S15–S16 Tables).

**Table 3:**
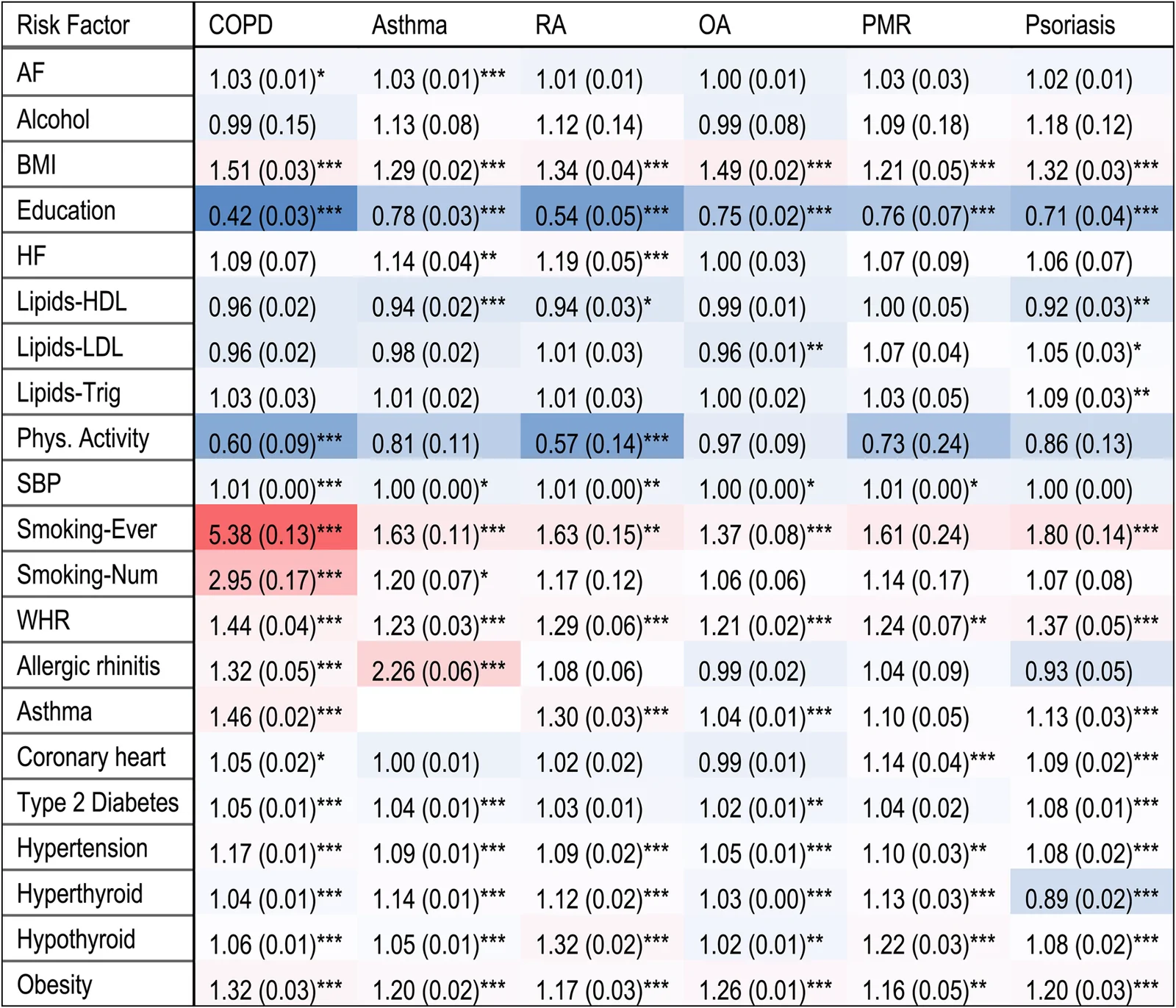
Risk factor causal effects on single diseases. Odds ratios (Standard Error) for risk factors affecting the single-disease outcome (inverse-variance–weighted MR). Estimates are OR = exp(β) reported as per 1-SD increase for continuous exposures and per 1-unit increase in genetic liability for binary liabilities (diseases). Detailed results are in Supplementary Table 11. Red denotes OR>1 and blue OR<1. *p* < 0.05; * *p* <0.01; ** *p* < 0.001

### Mendelian randomization

#### Asthma preceding a MSK condition

Using genetics, we found evidence that asthma could lead to RA (Table 1). Genetic liability to asthma was associated with increased risk of RA (OR per doubling of genetic liability = 1.19; 95% CI: 1.13–1.24; *p =* 1.02 × 10⁻^12^). This evidence remained robust in sensitivity analyses including radial MR and Steiger (Supplementary Table 8), which removed variants in *IL33*, *IL1RL1*, *TSLP*, *IL6R*, *HLA-DQA1* (Supplementary Material – Summary of instrument filtering, Supplementary Table 9–10). This evidence remained robust after excluding all HLA instruments, although with a weaker effect size: OR = 1.12; 95% CI 1.07–1.18; *p =* 1.981 × 10⁻⁶.The genetic evidence after excluding HLA instruments was less strong for other MSK conditions. Genetic liability to asthma was modestly associated with OA (OR = 1.03; 95% CI 1.01–1.05; *p =* 4.41 × 10⁻^4^), whereas there was no clear evidence for an effect on psoriasis (OR = 1.03; 95% CI 0.99–1.08; *p =* 0.103) or on PMR (OR = 1.03; 95% CI 0.96–1.10; *p =* 0.390). MVMR suggested partial attenuation through IL-6 (Supplementary Material – Comprehensive MR results, Supplementary Table 11).

#### MSK condition preceding asthma

Genetic analyses indicated that MSK conditions increase asthma risk. All MR estimates excluded HLA-region variants, except for PMR where insufficient non-HLA instruments were available. Genetic liability to OA was associated with an increased risk of asthma (OR = 1.14; 95% CI 1.08–1.19; *p =* 4.48 × 10⁻⁸). RA showed a comparable association with asthma (OR = 1.07; 95% CI 1.04–1.10; *p =* 1.86 × 10⁻⁶), and psoriasis demonstrated a weaker, directionally consistent effect (OR = 1.05; 95% CI 1.02–1.09; *p =* 6.99 × 10⁻⁶). There was no evidence of a causal effect of PMR on asthma.

#### COPD preceding MSK condition

Genetic liability to COPD was associated with an increased risk of RA (OR = 1.19; 95% CI 1.11–1.27; *p =* 3.93 × 10⁻⁷) and PMR (OR = 1.14; 95% CI 1.03–1.25; *p =* 9.89 × 10⁻^3^). Genetic liability to COPD also increased risk of OA (OR = 1.10; 95% CI 1.06–1.13; *p =* 4.00 × 10⁻⁸) and showed a smaller association with psoriasis (OR = 1.08; 95% CI 1.01–1.16; *p =* 3.51 × 10⁻^2^). MVMR showed partial attenuation from IL6 for the risk of COPD on RA and OA (ST11).

#### MSK condition preceding COPD

Genetic liability to RA was associated with an increased risk of COPD (OR = 1.10; 95% CI 1.07–1.12; *p =* 1.48 × 10⁻^14^). Liability to OA was also associated with higher COPD risk (OR = 1.19; 95% CI 1.13–1.25; *p =* 1.86 × 10⁻^11^); A weaker association was observed for psoriasis (OR = 1.04; 95% CI 1.02–1.07; *p =* 2.23 × 10⁻^3^) which was only significant after excluding the HLA region. The risk of PMR on COPD could not be estimated without HLA instruments.

### Observed evidence of co-occurrence

In observational data, asthma (median onset 44 years, CPRD) preceded musculoskeletal (MSK) diagnoses in most cases, occurring before RA (71.5%), OA (74.6%), and psoriasis (64.4%), with median intervals (time at which 50% of people in the multimorbid group had gotten the disease) of ~ 12–13 years In contrast, MSK diagnoses preceded asthma over shorter intervals (5–9 years) and were consistently associated with increased asthma risk (HRs ~ 1.3–1.5). For COPD, transitions were more rapid, with median intervals of ~ 4–6 years from COPD to MSK diagnoses and ~ 5–11 years in the reverse direction (see Figs. 1 and 2 for the mean intervals).

Observational analyses showed consistent bidirectional associations across all disease pairs (HRs ~ 1.1–1.9), generally stronger than the corresponding MR estimates. E-values for observational estimates ranged from ~ 1.3 to 3.2, indicating that while some associations may be susceptible to residual confounding, others—particularly COPD–RA—show greater robustness.

#### Prognosis of MSK and respiratory multimorbidity

Across six of the eight respiratory–musculoskeletal combinations, individuals with two conditions had higher 10-year risks of mortality and more hospitalisation in one year compared with those with the corresponding respiratory disease alone (all *p <* 0.001). The exception was pairs involving OA (Table 2).

For COPD multimorbidity, the greatest excess risk was observed for RA with subsequent COPD. Mortality was higher in the multimorbid group (HR 2.91; 95% CI 2.75–3.07; *p <* 0.001) than in those with COPD alone (HR 2.22; 95% CI 2.11–2.33; *p <* 0.001), and unplanned hospitalisations were also higher (IRR 2.54; 95% CI 2.37–2.73; *p <* 0.001 vs IRR 1.95; 95% CI 1.84–2.06; *p <* 0.001).

For asthma multimorbidity, the strongest effects were seen for RA with subsequent asthma. Mortality was greater in those with both conditions (HR 1.95; 95% CI 1.82–2.08; *p <* 0.001) than in those with asthma alone (HR 1.30; 95% CI 1.23–1.37; *p <* 0.001), and unplanned hospitalisations were also higher (IRR 2.22; 95% CI 2.05–2.41; *p <* 0.001 vs IRR 1.53; 95% CI 1.43–1.62; *p <* 0.001).

The order of diagnoses within the multimorbid groups generally made little difference to these prognostic estimates; where differences were observed, they were modest in size (HR/IRR differences ~ 0.1–0.2) and did not alter the overall pattern.

### Risk factors and mediators: candidate targets for intervention

#### Risk factors for MSK disease

Risk factor results are reported as ORs per 1 SD increase for continuous exposures and per 1-unit increase in genetic liability for disease.

Genetic liability to adiposity and smoking was consistently associated with higher MSK risk (BMI OR range 1.21–1.49, WHR 1.21–1.37, ever-smoking 1.37–1.80; all *p ≤* 0.01). The largest single effect was ever-smoking for psoriasis (OR 1.80; 95% CI 1.37–2.36; *p =* 2.47 × 10⁻^5^) (Table 3, S13). Higher educational attainment was protective across all four MSK outcomes (OR range 0.54–0.76; all *p <* 0.001). The strongest protective association was educational attainment for RA (OR 0.54; 95% CI 0.49–0.60; *p =* 5.41 × 10⁻^35^). Increasing HDL cholesterol was also protective for psoriasis (OR 0.92; 95% CI 0.86–0.98; *p =* 9.52 × 10⁻^3^), and higher Physical Activity (PA) was protective for RA (OR 0.57; 95% CI 0.44–0.74; *p =* 3.90 × 10⁻^5^).

Mediation analysis highlighted risk factors that become more relevant due to pre-existing disease and may speed up onset of a second associated disease (Fig. 3, S14). Mediation analyses show that reduced PA explains a proportion of the relationships between lung conditions leading to MSK conditions. Reduced PA in people with asthma partially explained the increased risk of OA (0.8%, *p <* 0.001), PMR (0.8%, *p =* 0.048) and psoriasis (0.7%, *p =* 0.012). Hypertension in people with asthma also mediated the development of PMR (3.2%, *p <* 0.001), while hypertension in people with COPD mediated a proportion of the risk of RA onset (0.1%, *p =* 0.004).

#### Risk factors for asthma

Statistically significant risk factors spanned adiposity, smoking, allergy, and cardiometabolic traits (significant OR range 1.05–2.26; e.g., BMI 1.29; WHR 1.23; ever-smoking 1.63; hypertension 1.09; hyperthyroidism 1.14) (Table 3, S13). The largest single effect was allergic rhinitis (OR 2.26; 95% CI 2.01–2.54; *p =* 1.69 × 10⁻^39^). Higher HDL levels (OR 0.94; 95% CI 0.91–0.98; *p =* 4.20 × 10⁻^4^) and education (OR 0.78; 95% CI 0.74–0.82; *p =* 3.27 × 10⁻^21^) were protective of onset of asthma.

Obesity and cholesterol became more important in the context of multimorbidity (Fig. 3, S14). Obesity and cholesterol mediated the observed effect of MSK conditions on the onset of asthma. LDL level (Low LDL, 0.1%, *p <* 0.001) and weight gain (obesity 2.7%, *p <* 0.001) in participants with OA partially mediated the risk of asthma onset. Similar effects were identified for psoriasis (obesity 1.4%, *p =* 0.004) and RA (high LDL, 1.1%, *p =* 0.032) for asthma onset. Lastly, allergic rhinitis in participants with OA explain 3.5% (*p =* 0.004) of the onset of asthma.

#### Risk factors for COPD

Statistically significant risk factors included smoking, adiposity, and cardiometabolic traits (e.g., ever-smoking 5.38, cigarettes/week 2.95, BMI 1.51, WHR 1.44, hypertension 1.17; all *p ≤* 0.001) (Table 3, S13). The largest single effect was ever smoking (OR 5.38; 95% CI 4.17–6.93; *p =* 1.18 × 10⁻^3^⁸). Protective factors included education and PA (OR range 0.42–0.60). The strongest protective factor was education (OR 0.42; 95% CI 0.39–0.45; *p =* 8.11 × 10⁻^145^).

Obesity became more relevant for COPD prevention in the context of pre-existing conditions (Fig. 3, S14). Obesity and cholesterol also mediated the causal effect of MSK conditions (except PMR) on onset of COPD [OA leading to COPD: low HDL 0.3%, *p <* 0.0001; RA leading to COPD: both low LDL (0.7%, *p =* 0.004) and high LDL (2.7%, *p =* 0.048); psoriasis leading to COPD: high triglycerides (0.6%, *p =* 0.024)]. Some cardiovascular diseases in participants with OA partially mediated the risk of COPD [coronary heart disease (3.7%, *p <* 0.0001); hypertension (0.4%, *p <* 0.0001)]. Limited PA in people with OA (3.5%, *p =* 0.004) and psoriasis (1.7%, *p =* 0.012) also mediated risk of COPD onset.

### Colocalisation and shared biology

Colocalisation analysis identified shared variants for three multimorbidity pairs (Supporting information, S15).

For asthma–psoriasis, a variant on chromosome 2 (rs2111485) between the *FAP* and *IFIH1* genes had strong evidence of colocalising for both conditions (region PPH4 = 0.98, SNP PPH4 = 0.97). The effect allele (rs2111485 A) had opposite directions of effect for risk of asthma (β = 0.031) and psoriasis (β = –0.066), compared to the G allele. The variant is not a known cis-pQTL but is a trans-QTL for BST2 (Bone marrow stromal antigen 2), linked to IFN-induced antiviral host response. rs2111485 has appeared in many previous GWAS of autoimmune and inflammatory conditions, in addition to strong haematology (white blood cell) count associations (GWAS cataolog www.ebi.ac.uk/gwas Nov 2025). This variant is in strong LD (R^2^ = 0.95) with rs1990760, a common *IFIH1* missense variant with a well-established link to type-1 diabetes [56]. Another region near the gene *IL7R* (region PPH4 = 0.99) showed weaker evidence for a specific variant (rs4594881, SNP PPH4 = 0.27) but with concordant directions.

For asthma–RA, the strongest signal was a variant (rs4938573) near the ~ 12kbp upstream of gene CXCR5 (region PPH4 = 0.94, SNP PPH4 = 1). rs4938573 is not a known pQTL but is a known eQTL for gene PHLDB1 (https://www.gtexportal.org/home/snp/rs4938573). UK Biobank participants carrying predicted loss-of-function variants in PHLDB1 had significantly reduced lung function (FEV1) and increased kidney diseases [57]. rs4938573 itself has appeared in a study in the GWAS catalog associated with neoplasm of mature B-cells [58]. There was weaker support evidence for variants in regions near ACOXL-AS1 on chromosome 2 (region PPH4 = 0.97, SNP PPH4 = 0.57 for rs77004761) and RUNX1 on chromosome 21 (region PPH4 = 0.97, SNP PPH4 = 0.53 for rs8129030).

For COPD–OA, rs429358 (in the *APOE* gene) had strong evidence of colocalisation (region PPH4 = 1.00, SNP PPH4 = 1 for rs429358), alongside weaker evidence near *BLK* on chromosome 8 (region PPH4 = 0.92, PPH4 = 0.12 for rs2409799). The *APOE* variant was found to be a cis-pQTL for APOE itself, and a *trans*-pQTL for 24 proteins including LEP (leptin), FBP1, and NCAN. Existing drugs targeting these proteins include lovastatin, gemfibrozil, and carvedilol (lipid and cardiovascular modulation), cyclosporine and tacrolimus (immunosuppressants), and valproic acid (neuroinflammation) (Supporting information, S16).

## Discussion

We used large genetic and EHR datasets to elucidate causal relationships between respiratory and musculoskeletal conditions, highlighting shared risk factors and diagnostic sequences. These findings provide insights into disease trajectories and suggest opportunities for preventive interventions to delay the onset of subsequent conditions.

MR suggested that some conditions share bidirectional relationships. Our findings support asthma as a causal factor for RA, consistent with higher rates of RA in individuals with childhood asthma [59]. The literature is sparse on evidence of RA causing asthma; however RA treatment may drive asthma exacerbations [60]. There is previous work showing COPD increases risk of RA [61]; Sero-positive RA in particular is related to COPD [62]. Partial attenuation after adjustment for IL-6 suggests that systemic inflammation contributes to the asthma → OA and COPD → RA relationships. IL-6 is an active mediator of airway disease and joint inflammation and is linked with the progression of all four diseases [63–65].

Other associations appeared unidirectional. MR identified for a link for the first time between COPD and the onset of PMR. A potential link could relate to COPD exacerbations as PMR has been observed to have a seasonal pattern [66]. Co-occurrence would usually prompt investigation for further diseases [67]. Further work will be needed to understand and verify the link. There was weak evidence for psoriasis being implicated in both lung conditions; psoriasis is known to associate with multiple conditions through pleiotropy [68].

Having both MSK and respiratory conditions is associated with higher mortality and hospitalisation rates, particularly for individuals with COPD and RA [69]. Preventing the onset of the second disease in an LTC pair provides opportunities for more targeted approaches that may extend survival and quality of life [69].

The median time between first and second diagnosis (the time by which 50% of the group had been diagnosed with the second disease) ranged from 5.5 years (COPD leading to PMR) to 13.2 years (asthma leading to PMR). Asthma often precedes the studied MSK conditions by over 10 years. Similarly, COPD often occurs an average of eight or more years after MSK conditions. These intervals reveal prolonged intervention windows to prevent progression to multimorbidity.

Shared risk factors across respiratory and MSK conditions included smoking status [70], obesity (e.g. BMI and waist-hip ratio) [71, 72], education level [73], and prior diagnosis of hypertension [74], hyperthyroidism and hypothyroidism. Thyroid dysfunction, including hyperthyroidism and hypothyroidism is known to be associated with respiratory impairment and MSK disorders [75, 76].

Mediation analyses showed that some risk factors emerged or gained importance in individuals diagnosed with the first disease of the pair, contributing to the onset of subsequent conditions. However, the estimated proportions mediated were small, indicating that most of the association likely operates through other pathways and these findings should be interpreted cautiously. Physical activity is an example. For pairs with asthma as the first condition, low physical activity, possibly due to avoidance of respiratory symptoms [77], explains part of its causal relationship with the incidence of PMR and psoriasis. Including exercise as part of treatment plans for asthma could improve asthma symptoms [78] and reduce the risk of some comorbidities [79]. People with asthma who regularly exercised developed OA faster, which may result from an interaction with a negative effect of corticosteroids on bone health and consequent wear and tear during activity [80]. Hypertension was a relatively strong mediator between asthma and PMR. Asthma has been found to increase the risk of primary hypertension; the replacement of long acting beta agonists with biologics has been suggested for individuals for whom asthma medications may have contributed to the onset of hypertension [81].

Among those with MSK conditions, some risk factors increased susceptibility to later respiratory disease LDL cholesterol emerged as a context-dependent mediator, with both low and high LDL contributing to respiratory multimorbidity. Low LDL in those with RA —consistent with the ‘RA lipid paradox’ [82]—mediated the onset of COPD. In RA, low LDL (often during active phases of the disease) reflects heightened systemic inflammation rather than favourable lipid status. Low LDL had been previously identified as a potential risk factor for COPD exacerbations [83]. A potential mechanism is reduced delivery of lipid-soluble antioxidants to airway tissues, promoting oxidative stress [84]. At the same time, high LDL also mediated COPD onset after RA, indicating that traditional cardiometabolic pathways may additionally contribute to respiratory risk in some individuals with RA. Obesity was an important factor in developing respiratory conditions after a MSK condition. Reduced mobility, chronic pain, and medications can lead to weight gain [85], which in turn can affect lung mechanics and general inflammation, triggering the onset of asthma and COPD [86].

We found genetic overlap between asthma and psoriasis, asthma and RA, and COPD and OA. We identified a shared causal variant between asthma–psoriasis near to gene *IFIH1* (rs2111485), where the A allele reduced risk for psoriasis yet increased risk of asthma [87]. *IFIH1* encodes protein MDA5 (melanoma differentiation-associated protein 5), involved in immune activation following virus detection. Our apparently discordant results may therefore reflect the antiviral role of MDA5, where variants that dampen interferon signalling can protect against autoimmune activation while increasing vulnerability to viral-triggered asthma [88]. The variant is co-inherited with a common MDA5 amino-acid-modifying variant (rs1990760) with a well-established link to type-1 diabetes [56]. For COPD and OA the known *APOE* e4 genetic variant (rs429358) had a high probability of being causal for both conditions. The variant is highly pleiotropic, affecting risk for Alzheimer’s disease, lipid levels, and cardiovascular traits. There are limited studies on many of the drugs associated with the shared protein pathways. In vitro evidence has shown valproic acid (normally an anti-epileptic) to reduce smoking-induced inflammation [89] and to repair damage in an inflammatory environment modelled on OA [90]. Future studies are needed to confirm the causal pathways, associated protein targets, and disease risk. There are also significant issues with valproate and teratogenicity, and use is highly restricted in those aged less than 55 years, which would limit its use clinically [91]. There was also some evidence for a colocalised variant in *BLK* (rs2409797). The variant is known to affect other proteins that are targets of existing drugs, such as fostamatinib. Fostamatinib is currently used to treat chronic immune thrombocytopenia, and some evidence shows success for treating OA in model organisms [92]. However, this drug is associated with numerous side effects [93]. Functional validation is required to confirm the mechanism linking the identified shared variant with causal mechanism, especially as the proteomic analysis could only study the 2923 plasma proteins included in UK Biobank.

This study has limitations related to data availability. The genetic analyses used publicly available GWAS of common genetic variants in participants of European genetic ancestry; exploration in other genetic ancestries was not possible, and the mechanisms identified may not generalize. Future work including other genetic ancestries and population representative data is required to get the full picture. Mendelian randomisation studies using instruments from the HLA region should be treated with caution given the extensive correlation between variants and with multiple conditions. The observed diagnosis trajectories may not accurately reflect patient experience of disease onset; there may be greater delays in diagnosis and accessing health care for MSK conditions compared to respiratory conditions. Due to limited data collection on risk factors in CPRD, the percentage mediated by risk factors is likely underestimated. The lack of granularity in smoking data means that the relationship between diagnosis with a disease and smoking behaviour often showed inconsistent mediation. In these cases, a mediation effect cannot be estimated even if present. There is likely to be residual confounding in observational analyses from unmeasured confounders. While directionally consistent, differences in effect sizes between CPRD and SIDIAP likely reflect known structural differences between databases, including coding granularity, healthcare system coverage, and population characteristics, rather than true biological inconsistency. The colocalisation analysis is exploratory, and the identified drug targets should be interpreted as indicative of underlying mechanisms rather than recommendations for clinical treatment.

We highlight potential windows in which specific MSK and respiratory multimorbidity sequences may be delayed, including the particular importance of reducing the co-occurrence of COPD and RA given the associated increase in mortality. Patients with established conditions such as RA —especially those with additional risk factors such as smoking, increased BMI, abnormal lipid profiles, or hypertension—may represent groups in whom more structured monitoring could be considered. For newly diagnosed RA, clinicians may wish to undertake baseline respiratory assessment, for example symptom review and spirometry (where clinically indicated), with repeat assessment in individuals who develop persistent respiratory symptoms or who have cardiovascular risk factors. Prospective studies are needed to determine whether periodic respiratory review, for example annually or aligned with routine RA follow-up, improves early detection of COPD.

Our identification of a potential link between COPD and subsequent PMR suggests that clinicians should remain alert to new musculoskeletal symptoms in patients with COPD, especially in older adults. The role of cardiometabolic factors as mediators between respiratory and MSK conditions supports a holistic approach to cardiovascular prevention, including attention to lipid management in RA and monitoring for medication-associated hypertension in asthma. Structured activity programmes, such as supervised exercise interventions tailored to disease-specific barriers including pain, instability or breathlessness, may help maintain physical function and could reduce the risk of multimorbidity progression [94]. For people already living with both types of conditions low impact modifications within pulmonary rehabilitation and physiotherapy programmes could be explored.

Overall, our findings indicate individuals diagnosed with a respiratory or MSK condition have increased genetic risk of a subsequent condition and represent a key target for intervention to delay multimorbidity. Further research is required to directly evaluate the effectiveness of specific preventive strategies during key intervention windows — both prior to and between the onset of diseases. Research that pinpoints interacting factors that predicts MLTC accumulation could help guide personalised prescribing. Future research could also test therapeutic targets along the pathways connecting shared risk factors such as smoking, BMI, and inflammation to cellular damage and ageing to determine whether they can prevent downstream disease [95].

## Supplementary Information

Supplementary Material 1.

Supplementary Material 2.

Supplementary Material 3.

Supplementary Material 4.

## Data Availability

The UK routine clinical data used in this study are available from CPRD. Access to CPRD data requires approval from the Medicines and Healthcare products Regulatory Agency’s Independent Scientific Advisory Committee (ISAC). The data are not publicly available due to licensing restrictions, but researchers may apply directly to CPRD at the following link: https://cprd.com/research-applications The UK Biobank data used in this research were accessed under Application 14,631. Researchers may apply for access to UK Biobank at: https://www.ukbiobank.ac.uk/enable-your-research The SIDIAP data used in this study are not publicly available. Access may be requested directly from SIDIAP at: https://www.sidiap.org/index.php/en/solicituds-en The GWAS summary statistics generated and/or used in this study, together with the diagnostic code lists used to define phenotypes, are openly available in our Zenodo repository: https://zenodo.org/records/14284047

## Abbreviations

BMI: Body mass index
CI: Confidence interval
COPD: Chronic obstructive pulmonary disease
CPRD: Clinical Practice Research Datalink
EHR: Electronic health record
GWAS: Genome-wide association study
HDL: High-density lipoprotein
HR: Hazard ratio
LDL: Low-density lipoprotein
MR: Mendelian randomisation
MSK: Musculoskeletal
OA: Osteoarthritis
OR: Odds ratio
PA: Physical activity
PMR: Polymyalgia rheumatica
pQTL: Protein quantitative trait locus
RA: Rheumatoid arthritis
SNP: Single-nucleotide polymorphism
