# Supplementary material for: Genetic and epidemiological evidence linking respiratory and musculoskeletal diseases: shared risk factors and intervention windows
Source: Respir Res. 2026 Jun 9;27:348. doi: 10.1186/s12931-026-03726-y (PMC13573401; doi:10.1186/s12931-026-03726-y)
Supplement: Supplementary file 1 [file 12931_2026_3726_MOESM2_ESM.pdf]

STROBE-MR checklist of recommended items to address in reports of Mendelian randomization studies[1 2]

Item No.	Section		Checklist item	Page No.	Relevant text from manuscript
1	**TITLE and ABSTRACT**		Indicate Mendelian randomization (MR) as the study's design in the title and/or the abstract if that is a main purpose of the study	2	Not in title as not the primary focus of the paper. Abstract Methods and Findings Paragraph 1
	**INTRODUCTION**				
2	**Background**		Explain the scientific background and rationale for the reported study. What is the exposure? Is a potential causal relationship between exposure and outcome plausible? Justify why MR is a helpful method to address the study question	5	Introduction: End of Paragraph 4
3	**Objectives**		State specific objectives clearly, including pre-specified causal hypotheses (if any). State that MR is a method that, under specific assumptions, intends to estimate causal effects	5	Introduction: End of Paragraph 4
	**METHODS**				
4	**Study design and data sources**		Present key elements of the study design early in the article. Consider including a table listing sources of data for all phases of the study. For each data source contributing to the analysis, describe the following:		
		a)	Setting: Describe the study design and the underlying population, if possible. Describe the setting, locations, and relevant dates, including periods of recruitment, exposure, follow-up, and data collection, when available.	5-6,	Methods: Data sources paragraphs 1 and 3: Described in more detail in Zenodo page, Murrin et al 2025 and Voller et al 2025
		b)	Participants: Give the eligibility criteria, and the sources and methods of selection of participants. Report the sample size, and whether any power or sample size calculations were carried out prior to the main analysis	5-6	Methods: Data sources paragraphs 2: Described in more detail in Zenodo page, Murrin et al 2025 and Voller et al 2025
		c)	Describe measurement, quality control and selection of genetic variants	Supplementary Material 3-4	Summary of instruments Filtering: Paragraphs 1:5, Additional Sensitivity Analysis Paragraph1
		d)	For each exposure, outcome, and other relevant variables, describe methods of assessment and diagnostic criteria for diseases	5	Methods:Data sources paragraph 1, Summary Stats derived from Murrin et al 2025
		e)	Provide details of ethics committee approval and participant informed consent, if relevant	7	Methods:Ethics paragraph 1
5	**Assumptions**		Explicitly state the three core IV assumptions for the main analysis (relevance, independence and exclusion restriction) as well assumptions for any additional or sensitivity analysis	Supplementary Material 2	MR Method Overview paragraph 5

6	**Statistical methods: main analysis**		Describe statistical methods and statistics used		
		a)	Describe how quantitative variables were handled in the analyses (i.e., scale, units, model)	7	Methods:Mendelian Randomisation paragraph 3
		b)	Describe how genetic variants were handled in the analyses and, if applicable, how their weights were selected	Supplementary material	MR Method Overview paragraph 2, Summary of instrument filtering whole section,S14,S15
		c)	Describe the MR estimator (e.g. two-stage least squares, Wald ratio) and related statistics. Detail the included covariates and, in case of two-sample MR, whether the same covariate set was used for adjustment in the two samples	Supplementary Material	MR Method Overview whole section
		d)	Explain how missing data were addressed	Supplementary Material	MR Method Overview: paragraph 2
		e)	If applicable, indicate how multiple testing was addressed		Not used as selecting a small number of conditions with prior genetic correlation and clinical evidence of association
7	**Assessment of assumptions**		Describe any methods or prior knowledge used to assess the assumptions or justify their validity	Supplementary Material	MR Method Overview whole section
8	**Sensitivity analyses and additional analyses**		Describe any sensitivity analyses or additional analyses performed (e.g. comparison of effect estimates from different approaches, independent replication, bias analytic techniques, validation of instruments, simulations)	Supplementary Material	MR Method Overview paragraph 6-7
9	**Software and pre-registration**				
		a)	Name statistical software and package(s), including version and settings used	Supplementary Material	MR Method Overview paragraph 5
		b)	State whether the study protocol and details were pre-registered (as well as when and where)		Not pre registered
	**RESULTS**				
10	**Descriptive data**				
		a)	Report the numbers of individuals at each stage of included studies and reasons for exclusion. Consider use of a flow diagram		
		b)	Report summary statistics for phenotypic exposure(s), outcome(s), and other relevant variables (e.g. means, SDs, proportions)		Reported in publications where summary statistics originate

		c)	If the data sources include meta-analyses of previous studies, provide the assessments of heterogeneity across these studies		
		d)	For two-sample MR:<br>i. Provide justification of the similarity of the genetic variant-exposure associations between the exposure and outcome samples<br>ii. Provide information on the number of individuals who overlap between the exposure and outcome studies	5-6	Methods: Data Sources paragraphs 1–3.<br>Complete overlap between diseases, very little overlap between risk factor and disease cohorts
11	**Main results**				
		a)	Report the associations between genetic variant and exposure, and between genetic variant and outcome, preferably on an interpretable scale		S15
		b)	Report MR estimates of the relationship between exposure and outcome, and the measures of uncertainty from the MR analysis, on an interpretable scale, such as odds ratio or relative risk per SD difference	14,15,17,18	[Disease] preceding [disease] paragraph 1 of each<br>Risk Factors for [Disease] paragraph 1 of each
		c)	If relevant, consider translating estimates of relative risk into absolute risk for a meaningful time period		Not applicable as absolute risks cannot be estimated from summary-level MR and disease incidence varies across contributing cohorts
		d)	Consider plots to visualize results (e.g. forest plot, scatterplot of associations between genetic variants and outcome versus between genetic variants and exposure)	Supplementary Material	Further Sensitivity analyses figure S1
12	**Assessment of assumptions**				
		a)	Report the assessment of the validity of the assumptions	14, Supplementary Material	Asthma preceding MSK, Supplementary Material: Comprehensive MR results
		b)	Report any additional statistics (e.g., assessments of heterogeneity across genetic variants, such as I^2, Q statistic or E-value)	14, Supplementary Material	Asthma preceding MSK, Supplementary Material: Comprehensive MR results
13	**Sensitivity analyses and additional analyses**				
		a)	Report any sensitivity analyses to assess the robustness of the main results to violations of the assumptions	Supplementary Material	Comprehensive MR results

		b)	Report results from other sensitivity analyses or additional analyses	Supplementary Material	Further Sensitivity Analyses
		c)	Report any assessment of direction of causal relationship (e.g., bidirectional MR)	Supplementary Material	Comprehensive MR results
		d)	When relevant, report and compare with estimates from non-MR analyses	14 15,16,17	[Disease] preceding [disease] paragraph 2 of each<br>Risk Factors for [Disease] paragraph 2 of each
		e)	Consider additional plots to visualize results (e.g., leave-one-out analyses)		Not reported
	**DISCUSSION**				
14	**Key results**		Summarize key results with reference to study objectives	21	Discussion: Paragraphs, 2,3
15	**Limitations**		Discuss limitations of the study, taking into account the validity of the IV assumptions, other sources of potential bias, and imprecision. Discuss both direction and magnitude of any potential bias and any efforts to address them	24	Discussion Paragraph 10
16	**Interpretation**				
		a)	Meaning: Give a cautious overall interpretation of results in the context of their limitations and in comparison with other studies	21	Discussion: Paragraphs, 2,3
		b)	Mechanism: Discuss underlying biological mechanisms that could drive a potential causal relationship between the investigated exposure and the outcome, and whether the gene-environment equivalence assumption is reasonable. Use causal language carefully, clarifying that IV estimates may provide causal effects only under certain assumptions	21,22	Discussion paragraph 3,7
		c)	Clinical relevance: Discuss whether the results have clinical or public policy relevance, and to what extent they inform effect sizes of possible interventions	21,22,24,25	Discussion paragraph 1,4,11,12
17	**Generalizability**		Discuss the generalizability of the study results (a) to other populations, (b) across other exposure periods/timings, and (c) across other levels of exposure	20	Discussion paragraph 10
	**OTHER INFORMATION**				
18	**Funding**		Describe sources of funding and the role of funders in the present study and, if applicable, sources of funding for the databases and original study or studies on which the present study is based	26	Funding
19	**Data and data sharing**		Provide the data used to perform all analyses or report where and how the data can be accessed, and reference these sources in the article. Provide the	25	Data availability

		statistical code needed to reproduce the results in the article, or report whether the code is publicly accessible and if so, where		
20	**Conflicts of Interest**	All authors should declare all potential conflicts of interest	28	Declaration of Interests

This checklist is copyrighted by the Equator Network under the Creative Commons Attribution 3.0 Unported (CC BY 3.0) license.

1. Skrivankova VW, Richmond RC, Woolf BAR, Yarmolinsky J, Davies NM, Swanson SA, et al. Strengthening the Reporting of Observational Studies in Epidemiology using Mendelian Randomization (STROBE-MR) Statement. JAMA. 2021;under review.

2. Skrivankova VW, Richmond RC, Woolf BAR, Davies NM, Swanson SA, VanderWeele TJ, et al. Strengthening the Reporting of Observational Studies in Epidemiology using Mendelian Randomisation (STROBE-MR): Explanation and Elaboration. BMJ. 2021;375:n2233.
