# Supplementary material for: Genetic and epidemiological evidence linking respiratory and musculoskeletal diseases: shared risk factors and intervention windows
Source: Respir Res. 2026 Jun 9;27:348. doi: 10.1186/s12931-026-03726-y (PMC13573401; doi:10.1186/s12931-026-03726-y)
Supplement: Supplementary file 2 [file 12931_2026_3726_MOESM3_ESM.pdf]

The RECORD statement – checklist of items, extended from the STROBE statement, that should be reported in observational studies using routinely collected health data.

	Item No.	STROBE items	Location in manuscript where items are reported	RECORD items	Location in manuscript where items are reported
**Title and abstract**					
	1	(a) Indicate the study's design with a commonly used term in the title or the abstract<br><br>(b) Provide in the abstract an informative and balanced summary of what was done and what was found	Pg 2<br><br>Pg 2	RECORD 1.1: The type of data used should be specified in the title or abstract. When possible, the name of the databases used should be included.<br><br>RECORD 1.2: If applicable, the geographic region and timeframe within which the study took place should be reported in the title or abstract.<br><br>RECORD 1.3: If linkage between databases was conducted for the study, this should be clearly stated in the title or abstract.	Abstract: Methods and Findings: Paragraph 1<br><br>Abstract: Methods and Findings: Paragraph 1 (UK EHR, CPRD, age ≥40, N=11M)<br><br>No New linkage
**Introduction**					
Background rationale	2	Explain the scientific background and rationale for the investigation being reported	Pg 4-5		Introduction paragraph 1-5
Objectives	3	State specific objectives, including any prespecified hypotheses	Pg 5		Introduction paragraph 5
**Methods**					
Study Design	4	Present key elements of study design early in the paper	Pg 5–7 (Introduction approach + Observational design described)		
Setting	5	Describe the setting, locations, and relevant dates, including	Methods: Data sources paragraph 3 (pg 6)		

		periods of recruitment, exposure, follow-up, and data collection			
Participants	6	*(a) Cohort study* - Give the eligibility criteria, and the sources and methods of selection of participants. Describe methods of follow-up<br><br>*(b) Cohort study* - For matched studies, give matching criteria and number of exposed and unexposed	Methods: Data sources paragraph 3 (pg 6)<br><br>Observational analysis Pg 8 paragraph 3 (Supporting Information Tables S3–S4).	RECORD 6.1: The methods of study population selection (such as codes or algorithms used to identify subjects) should be listed in detail. If this is not possible, an explanation should be provided.<br><br>RECORD 6.2: Any validation studies of the codes or algorithms used to select the population should be referenced. If validation was conducted for this study and not published elsewhere, detailed methods and results should be provided.<br><br>RECORD 6.3: If the study involved linkage of databases, consider use of a flow diagram or other graphical display to demonstrate the data linkage process, including the number of individuals with linked data at each stage.	Methods: Data sources paragraph 1 (pg 5)<br><br>Methods: Data sources paragraph 1 (pg 5) Murrin *et al.* 2025<br><br>NA
Variables	7	Clearly define all outcomes, exposures, predictors, potential confounders, and effect modifiers. Give diagnostic criteria, if applicable.	Methods: Data sources paragraph 1,3,4 (pg 5-6)<br><br>Supporting Information Tables (S1–S2).	RECORD 7.1: A complete list of codes and algorithms used to classify exposures, outcomes, confounders, and effect modifiers should be provided. If these cannot be reported, an explanation should be provided.	Codelist for diseases (exposure and outcome) Releases · GEMINI-multimorbidity/GEMINI-LTC-code-list-Public (github.com)

					(https://github.com/GEMINI-multimorbidity/GEMINI-LTC-code-list-Public).<br><br>Effect modifiers: S2
Data sources/ measurement	8	For each variable of interest, give sources of data and details of methods of assessment (measurement).<br>Describe comparability of assessment methods if there is more than one group	Data sources: Pargraphs: 1,4 (pg 5-6)<br><br>Prognostic consequences: (whole section) pg 9		
Bias	9	Describe any efforts to address potential sources of bias	Matching process: pg 8-9<br>Confounders adjusted for: Supplementary table 2		
Study size	10	Explain how the study size was arrived at	Methods Data Sources paragraph 3, pg 6		
Quantitative variables	11	Explain how quantitative variables were handled in the analyses. If applicable, describe which groupings were chosen, and why	Supplementary table 2 (clinically relevant cut offs)		
Statistical methods	12	(a) Describe all statistical methods, including those used to control for confounding<br>(b) Describe any methods used to examine subgroups and interactions	a) Methods: Observational analyses paragraphs pg 8-11		

		(c) Explain how missing data were addressed<br>(d) *Cohort study* - If applicable, explain how loss to follow-up was addressed<br>(e) Describe any sensitivity analyses	b) None<br>c)Methods Data sources last paragraph, pg 6;<br>d) deregistration (Observational Analyses paragraph 2 ,pg 8)<br><br>e) None		
Data access and cleaning methods		..		RECORD 12.1: Authors should describe the extent to which the investigators had access to the database population used to create the study population.<br><br>RECORD 12.2: Authors should provide information on the data cleaning methods used in the study.	Author contributions; pg 27.
Linkage		..		RECORD 12.3: State whether the study included person-level, institutional-level, or other data linkage across two or more databases. The methods of linkage and methods of linkage quality evaluation should be provided.	No Linkage
**Results**					
Participants	13	(a) Report the numbers of individuals at each stage of the study (*e.g.*, numbers potentially eligible, examined for eligibility, confirmed eligible, included in the study, completing follow-up, and analysed)	Supplementary Information Observational Note	RECORD 13.1: Describe in detail the selection of the persons included in the study (*i.e.,* study population selection) including filtering based on data quality, data availability and linkage. The selection of included persons can	Supplementary Information Observational Note

		(b) Give reasons for non-participation at each stage.<br>(c) Consider use of a flow diagram		be described in the text and/or by means of the study flow diagram.	
Descriptive data	14	(a) Give characteristics of study participants (*e.g.*, demographic, clinical, social) and information on exposures and potential confounders<br>(b) Indicate the number of participants with missing data for each variable of interest<br>(c) *Cohort study* - summarise follow-up time (*e.g.*, average and total amount)	a) Supplementary table 3:5<br><br>b) Supplementary table 3:5<br><br>c)10 years (prognostic consequences: pg 9) Supplementary Material Observational Note		
Outcome data	15	*Cohort study* - Report numbers of outcome events or summary measures over time	Supplementary table 3:5		
Main results	16	(a) Give unadjusted estimates and, if applicable, confounder-adjusted estimates and their precision (e.g., 95% confidence interval). Make clear which confounders were adjusted for and why they were included<br>(b) Report category boundaries when continuous variables were categorized<br>(c) If relevant, consider translating estimates of relative	a) Tables 1:2<br><br>b) Supplementary table 2<br><br>c) Not done		

		risk into absolute risk for a meaningful time period			
Other analyses	17	Report other analyses done—e.g., analyses of subgroups and interactions, and sensitivity analyses	Not done		
**Discussion**					
Key results	18	Summarise key results with reference to study objectives	paragraphs 1,4-8 pg 21-23		
Limitations	19	Discuss limitations of the study, taking into account sources of potential bias or imprecision. Discuss both direction and magnitude of any potential bias	Discussion, paragraph 10 pg 24	RECORD 19.1: Discuss the implications of using data that were not created or collected to answer the specific research question(s). Include discussion of **misclassification bias, unmeasured confounding, missing data,** and changing eligibility over time, as they pertain to the study being reported.	Discussion, paragraph 10 pg 24
Interpretation	20	Give a cautious overall interpretation of results considering objectives, limitations, multiplicity of analyses, results from similar studies, and other relevant evidence	Discussion, paragraphs 1–4 and 11–12 pg 21,24-25		
Generalisability	21	Discuss the generalisability (external validity) of the study results	Discussion, paragraphs 10 and 12 pg 24-25		
**Other Information**					
Funding	22	Give the source of funding and the role of the funders for the present study and, if applicable, for the original study on which the present article is based	Funding pg 26		
Accessibility of protocol, raw data, and		..		RECORD 22.1: Authors should provide information on how to access any supplemental information such as	Data Availability Pg 25

programming code				the study protocol, raw data, or programming code.	

*Reference: Benchimol EI, Smeeth L, Guttmann A, Harron K, Moher D, Petersen I, Sørensen HT, von Elm E, Langan SM, the RECORD Working Committee. The REporting of studies Conducted using Observational Routinely-collected health Data (RECORD) Statement. *PLoS Medicine* 2015; in press.

*Checklist is protected under Creative Commons Attribution (CC BY) license.
