# Supplementary material for: Genetic and epidemiological evidence linking respiratory and musculoskeletal diseases: shared risk factors and intervention windows
Source: Respir Res. 2026 Jun 9;27:348. doi: 10.1186/s12931-026-03726-y (PMC13573401; doi:10.1186/s12931-026-03726-y)
Supplement: Supplementary file 3 [file 12931_2026_3726_MOESM1_ESM.docx]

Supplementary Information

Contents

[Risk Factor GWAS sources	2](#_Toc226662291)

[Propensity Score Covariates	2](#_Toc226662292)

[MR Method Overview	4](#_Toc226662293)

[Summary of Instrument filtering	5](#_Toc226662294)

[Further Sensitivity analyses	6](#_Toc226662295)

[Comprehensive MR results	8](#_Toc226662296)

[Observational Note on Coding	15](#_Toc226662303)

[Strobe MR	16](#_Toc226662304)

[Observational RECORD Checklist	16](#_Toc226662305)

[Suplementary Table Captions	16](#_Toc226662306)

[**Supplementary Table 1.**	16](#_Toc226662307)

[**Supplementary Table 2.**	16](#_Toc226662308)

[**Supplementary Table 3.**	16](#_Toc226662309)

[**Supplementary Table 4.**	16](#_Toc226662310)

[**Supplementary Table 5.**	17](#_Toc226662311)

[**Supplementary Table 6.**	17](#_Toc226662312)

[**Supplementary Table 7.**	17](#_Toc226662313)

[**Supplementary Table 8.**	17](#_Toc226662314)

[**Supplementary Table 9.**	17](#_Toc226662315)

[**Supplementary Table 10.**	17](#_Toc226662316)

[**Supplementary Table 11.**	17](#_Toc226662317)

[**Supplementary Table 12**	18](#_Toc226662318)

[**Supplementary Table 13.**	18](#_Toc226662319)

[**Supplementary Table 14.**	18](#_Toc226662320)

[**Supplementary Table 15.**	18](#_Toc226662321)

[**Supplementary Table 16.**	18](#_Toc226662322)

[References	19](#_Toc226662323)

# Risk Factor GWAS sources

- BMI [1]
- WHR [2]
- Cholesterol [3] including:
  - LDL
  - HDL
  - Triglycerides
- Educational attainment [4]
- Smoking status [5] including:
  - Ever smoked
  - Smoking intensity in smokers
- Alcohol consumption [5]
- Physical activity [6]

## Propensity Score Covariates

(Observational disease pair analysis and mediation)

For the asthma–PMR pair, the selected covariates are: IMD, LDL, HDL, BMI, total cholesterol, smoking status, physical activity level, triglycerides, alcohol consumption, albumin, ethnicity, region (East, South-East, West), allergic rhinitis, and atopic dermatitis.

For the asthma–psoriasis pair, the selected covariates are: IMD, LDL, HDL, BMI, total cholesterol, smoking status, physical activity level, triglycerides, alcohol consumption, albumin, ethnicity, region (East, South-East, West), allergic rhinitis, atopic dermatitis, coronary disease, and type 2 diabetes.

For the asthma–RA pair, the selected covariates are: IMD, LDL, HDL, BMI, total cholesterol, smoking status, physical activity level, triglycerides, alcohol consumption, albumin, ethnicity, region (East, South-East, West), allergic rhinitis, coronary disease, and type 2 diabetes.

For the COPD–gout pair, the selected covariates are: IMD, LDL, HDL, BMI, total cholesterol, smoking status, physical activity level, triglycerides, alcohol consumption, albumin, ethnicity, region (East, South-East, West), allergic rhinitis, atopic dermatitis, coronary disease, and type 2 diabetes.

For the COPD–OA pair, the selected covariates are: IMD, LDL, HDL, BMI, total cholesterol, smoking status, physical activity level, triglycerides, alcohol consumption, albumin, ethnicity, region (East, South-East, West), allergic rhinitis, atopic dermatitis, coronary disease, and type 2 diabetes.

For the COPD–osteoporosis pair, the selected covariates are: IMD, LDL, HDL, BMI, total cholesterol, smoking status, physical activity level, triglycerides, alcohol consumption, albumin, ethnicity, region (East, South-East, West), allergic rhinitis, atopic dermatitis, coronary disease, and type 2 diabetes.

For the COPD–PMR pair, the selected covariates are: IMD, LDL, HDL, BMI, total cholesterol, smoking status, physical activity level, triglycerides, alcohol consumption, albumin, ethnicity, region (East, South-East, West), allergic rhinitis, and atopic dermatitis.

For the COPD–psoriasis pair, the selected covariates are: IMD, LDL, HDL, BMI, total cholesterol, smoking status, physical activity level, triglycerides, albumin, ethnicity, region (East, South-East, West), allergic rhinitis, atopic dermatitis, and coronary disease.

For the gout–COPD pair, the selected covariates are: IMD, LDL, HDL, BMI, total cholesterol, smoking status, physical activity level, triglycerides, alcohol consumption, albumin, ethnicity, region (East, South-East, West), atopic dermatitis, coronary disease, and type 2 diabetes.

For the OA–COPD pair, the selected covariates are: IMD, LDL, HDL, BMI, total cholesterol, smoking status, physical activity level, triglycerides, alcohol consumption, albumin, ethnicity, region (East, South-East, West), allergic rhinitis, atopic dermatitis, coronary disease, and type 2 diabetes.

For the osteoporosis–COPD pair, the selected covariates are: IMD, LDL, HDL, BMI, total cholesterol, smoking status, physical activity level, triglycerides, alcohol consumption, albumin, ethnicity, region (East, South-East, West), and coronary disease.

For the PMR–asthma pair, the selected covariates are: IMD, LDL, HDL, systolic blood pressure, BMI, total cholesterol, smoking status, physical activity level, triglycerides, albumin, ethnicity, region (East, South-East, West), allergic rhinitis, atopic dermatitis, and coronary disease.

For the PMR–COPD pair, the selected covariates are: IMD, LDL, HDL, BMI, total cholesterol, smoking status, physical activity level, triglycerides, alcohol consumption, albumin, ethnicity, region (East, South-East, West), atopic dermatitis, and coronary disease.

For the psoriasis–asthma pair, the selected covariates are: IMD, LDL, HDL, BMI, total cholesterol, smoking status, physical activity level, triglycerides, alcohol consumption, albumin, ethnicity, region (East, South-East, West), allergic rhinitis, atopic dermatitis, coronary disease, and type 2 diabetes.

For the psoriasis–COPD pair, the selected covariates are: IMD, LDL, HDL, BMI, total cholesterol, smoking status, physical activity level, triglycerides, alcohol consumption, albumin, ethnicity, region (East, South-East, West), allergic rhinitis, atopic dermatitis, coronary disease, and type 2 diabetes.

For the RA–asthma pair, the selected covariates are: IMD, LDL, HDL, BMI, smoking status, physical activity level, triglycerides, alcohol consumption, albumin, ethnicity, region (East, South-East, West), allergic rhinitis, and coronary disease.

# MR Method Overview

summary statistics from meta-analysed cohorts available from (<https://zenodo.org/records/14284047>) were used to test the hypothesis that breathing conditions and rheumatic conditions might be causally associated.

Genetic variants that were strongly associated with the exposure (p<5×10⁻⁸), common (minor allele frequency [MAF] >0.1%), and independent (R^2^<0.01 in unrelated UK Biobank European-like participants). Missing data were handled during harmonisation; exposure variants unavailable in the outcome GWAS or failing allele alignment were excluded from the instrument set.

Whilst instrumenting COPD regions surrounding smoking genes and LD associated SNPS were manually excluded (e.g., CHRNA5 cluster on chromosome 15 behaviour and the DBH gene as these are linked to smoking). We did this because smoking is such a strong cause of COPD whilst also being a risk factor for many conditions.

To assess the potential causal effects of asthma and COPD on the risk of developing rheumatic conditions such as rheumatoid arthritis (RA) and polymyalgia rheumatica (PMR), we first harmonised GWAS summary statistics and We used RadialMR outlier detection to identify and exclude instruments with evidence of horizontal pleiotropy or excessive heterogeneity. We also ran Steiger filtering to remove SNPs that showed a higher r squared for the outcome than the exposure which could suggest pleiotropy and reverse causality.

We used TwoSampleMR R package v0.6.15 to estimate associations between exposure and outcome GWAS estimates. The primary estimate is from an Inverse Variance Weighted (IVW) regression that combines the ratio estimates for the individual instruments, weighted by the variance in the estimate. The IVW estimate is the most efficient (well-powered) estimate *when all genetic variants are valid instruments*. MR analysis has three core assumptions: 1) the relevance assumption (the genetic variants are strongly associated with the exposure disease), 2) the independence assumption (the genetic variants are not associated with confounders of the exposure-outcome relationship), and 3) the exclusion restriction assumption (the genetic variants only affect the outcome disease through the exposure disease).

MR was performed using multiple methods, including inverse variance weighted (IVW), MR-Egger, weighted median, weighted mode, and MR-GRIP. We assessed heterogeneity (Cochran’s Q), horizontal pleiotropy (Egger intercept), and instrument strength (F-statistic). Results were visualized using scatter, forest, and leave-one-out plots. Additionally, custom plots highlighted SNPs in the HLA region to evaluate whether these regions disproportionately influenced causal estimates. Bidirectional analyses were conducted for all primary traits, and results were saved with and without exclusion of SNPs in the HLA region (chr6:25–34Mb).

As a sensitivity analysis, we applied MVMR to estimate the direct causal effect of potentially correlated risk factors on each outcome and to evaluate whether bidirectional MR effects between respiratory and MSK conditions persisted after adjusting for these exposures. Instruments were clumped and harmonized across all exposures; we used Q-het (Q-minimization) to reduce bias from conditionally weak instruments (a significant issue for COPD and asthma in UK Biobank due to the UKBiLEVE recruitment criteria) and balanced pleiotropy, incorporated a phenotypic covariance matrix for exposures, and performed radial MVMR to remove pleiotropic outliers. We additionally repeated analyses after excluding HLA (chr6:25–34 Mb) and known smoking-related regions for COPD models.

Summary of Instrument filtering

For the asthma-to-rheumatic and skin condition analyses (including RA, PMR, OA, and psoriasis), several genetic variants were removed due to potential pleiotropy or reverse causality. Multiple asthma instruments mapped to the *HLA-DQA1* region, a known immunogenetic hotspot. Additional variants located in or near *IL33*, *ZPBP2*, and *TSLP*—genes involved in immune regulation—were also excluded due to broad pleiotropic effects across inflammatory pathways. In total, over 25 SNPs were removed across the asthma-related models. Among these, rs4916514 (located in *RERE*) was specifically flagged in the asthma → RA direction by Steiger filtering, as it explained more variance in RA than in asthma.

For the COPD analyses, including its associations with asthma, RA, PMR, psoriasis, and OA, instruments were filtered a priori to avoid variants strongly linked to smoking behaviour. Key regions excluded a priori included the *CHRNA5–CHRNA3* cluster on chromosome 15 and *DBH*.. Steiger filtering identified further variants as potentially invalid, including SNPs near *HLA-DQA1*, *NPNT*, and *INTS12*, which are involved in both pulmonary and systemic immune functions.

In the rheumatoid arthritis models assessing effects on asthma and COPD, several outliers were detected and removed. These included SNPs in *ZFP57*, *IRF5*, and *PVT1*.

Polymyalgia rheumatica presented challenges due to the limited number of genome-wide significant SNPs available. Most of the few instruments that passed genome-wide significance thresholds mapped to pleiotropic or immune-related loci. Variants in regions such as *HLA*, *ANKRD55*, and *PHF1* were flagged as outliers, and several were excluded either through heterogeneity filtering or directionality checks.

For osteoarthritis and psoriasis in relation to asthma and COPD, instruments were impacted by variants in regions with known immune and metabolic pleiotropy. SNPs in *APOE*, *TOMM40*, *HLA-DRA*, and *MIR1915* were among those filtered due to evidence of pleiotropy or directional inconsistency. One notable example was rs12357321 in *CASC10*, which was excluded from the OA models by radial filtering due to its broad associations with immune and metabolic traits.

The complete list of SNPs filtered out during analysis can be found in Supplementary tables **S14** The final SNP instruments used and their effect sizes for each exposure outcome pair can be found in **S15**.

# Further Sensitivity analyses

On inspection of scatter plots there were remaining SNPs in the HLA regions that had not been filtered by radial or steiger methods which seemed to have differential effects. For example the HLA region was increasing the overall estimation of effect for asthma -> Psoriasis (figure S1) but showed non significant protective effects for COPD in RA -> COPD (figure s2). Therefore where possible a second “No HLA” analysis was ran. MVMR was used to assess the impact of asthma and BMI as a confounder within pairs of genetic analyses. MR results with and without the HLA region intact were performed to check that any relationship was not purely driven by the HLA regions which can be pleiotropic

Figure s‑1 scatter plot for RA -> COPD with HLA regions highlighted in red

Figure s‑2 scatter plot for asthma -> Psoriasis with HLA regions highlighted in red

# Comprehensive MR results

**Causal estimates reported as the odds of developing the outcome disease during lifetime based on a doubling of genetic liability to the exposure disease** **Multivariable Mendelian randomization results were interpreted primarily using weak-instrument-robust Qhet estimates; attenuation is only reported where supported by Qhet analyses**

COPD _-> MSK

**COPD → Psoriasis**

In the raw IVW analysis, genetic liability to COPD was associated with an increased risk of psoriasis (OR = 1.08; 95% CI: 1.01–1.16; p = 0.033), though moderate heterogeneity was present (Q = 72.2, DF = 40; p = 0.0014). Radial MR removed one outlier SNP in a non-coding RNA (ncRNA) region, reducing heterogeneity (Q = 64.0, DF = 39; p = 0.0072), while preserving the IVW estimate (OR = 1.08; 95% CI: 1.01–1.15; p = 0.035). Steiger filtering retained this weak association after removing a SNP in the HLA region **(OR = 1.08; 95% CI: 1.01–1.15; p = 0.035**). Other MR methods showed broadly consistent results at this final stage: the weighted median (OR = 1.08; p = 0.061) and MR GRIP (OR = 1.18; p = 0.060) supported a similar direction of effect, though MR Egger remained non-significant. Instrument strength was acceptable (mean F-statistic = 37.6). MR without the HLA regions was the same as HLA variants were already removed as outliers by Radial MR and steiger filtering. This weak association was separate from the effect of asthma according to MVMR.

**COPD → Osteoarthritis (OA)**

Initial IVW MR suggested a moderate effect of genetic liability to COPD on osteoarthritis risk (OR = 1.14; 95% CI: 1.08–1.22; p = 6.8×10⁻⁵), though this was accompanied by substantial heterogeneity (Q = 205.6, DF = 40; p < 1×10⁻²²). Radial MR removed six outliers that were likely pleiotropic APOE, NPNT, and FAM13A regions ,reducing heterogeneity (Q = 78.2, DF = 34; p = 2.5×10⁻⁵) and yielding a more stable estimate (OR = 1.10; 95% CI: 1.06–1.13; p = 1.78×10⁻⁸).

**After Steiger filtering, the final IVW estimate remained robust** (**OR = 1.10; 95% CI: 1.06–1.13; p = 1.78×10⁻⁸**), supporting a directional effect from COPD to OA. The weighted median estimate was consistent (OR = 1.08; p = 8.5×10⁻⁵), and although MR Egger showed no significant association (OR = 1.02; p = 0.85), there was no evidence of directional pleiotropy (Egger intercept p = 0.39). MR without the HLA regions was the same as HLA variants were already removed as outliers by Radial MR and steiger filtering. The F statistic was acceptable.

Multivariable MR suggested that BMI may account for approximately 50% of this relationship in conventional analyses; however, this attenuation was not supported in weak-instrument-robust Qhet analyses.

**COPD → Polymyalgia Rheumatica (PMR)**

Initial inverse-variance weighted (IVW) Mendelian randomization suggested a positive association between genetic liability to COPD and increased risk of polymyalgia rheumatica (PMR), with an odds ratio of 1.23 (95% CI: 1.07–1.42; p = 4.1×10⁻³). This was accompanied by moderate heterogeneity (Q = 92.7, DF = 40; p = 4.5×10⁻⁶). Sensitivity analyses supported the IVW findings to varying degrees. The weighted median method produced a similar effect estimate (OR = 1.18; 95% CI: 1.03–1.36; p = 0.016), while MR Egger returned a wider and non-significant confidence interval (OR = 1.30; 95% CI: 0.72–2.37; p = 0.391), and the MR Egger intercept suggested no evidence of directional pleiotropy (p = 0.85).

Outlier correction using Radial MR improved model fit (Q = 38.5, DF = 38; p = 0.45) and yielded a more conservative yet still significant IVW estimate (OR = 1.14; 95% CI: 1.03–1.25; p = 9.9×10⁻³). Supporting methods at this stage remained directionally consistent, although several were no longer statistically significant.

**After Steiger filtering to retain only directionally valid instruments, the final IVW estimate remained significant and consistent (OR = 1.14; 95% CI: 1.03–1.25; p = 9.9×10⁻³).** Instrument strength was strong, with a mean F-statistic of 37.6, suggesting minimal risk of weak instrument bias.

MR without the HLA regions was the same as HLA variants were already removed as outliers by Radial MR and steiger filtering.

In multivariable MR analyses adjusting for asthma liability, attenuation of the COPD → PMR association was observed in conventional IVW models but was not supported by weak-instrument-robust Qhet estimates, suggesting this attenuation likely reflects instrument instability or shared genetic architecture rather than a robust mediating effect.

**COPD → Rheumatoid Arthritis (RA)**

The original IVW analysis indicated a strong association (OR = 1.48; 95% CI: 1.08–2.02; p = 0.016) but showed extreme heterogeneity (Q = 1020, DF = 40; p < 1×10⁻¹⁸).

Radial MR removed two outliers one in the NPNT region and a HLA-DQA1 variant which had been driving a lot of the heterogeneity, reducing heterogeneity (Q = 77.7, DF = 38; p = 0.036) and slightly attenuating the estimate (OR = 1.30; 95% CI: 1.00–1.69; p = 0.047). Steiger filtering removed another HLA-DQA1 variant and yielded a stable and robust IVW effect (**OR = 1.20; 95% CI: 1.12–1.28; p = 2.8×10⁻⁸),** with consistency across methods: weighted median (OR = 1.13; p = 7.5×10⁻⁴), weighted mode (OR = 1.08; p = 0.215), and MR GRIP (OR = 1.12; p = 0.187). MR Egger remained non-significant, and no evidence of directional pleiotropy was found (intercept p = 0.35). Instruments were strong (mean F = 37.6).

MR without the HLA regions was very similar as HLA variants were already removed as outliers by Radial MR and Steiger filtering.

Multivariable MR analyses indicated partial attenuation of this association after adjustment for IL6, and this effect persisted under weak-instrument-robust Qhet estimates, supporting a role for systemic inflammation in mediating part of the COPD → RA relationship

# MSK -> COPD

**Psoriasis → COPD**

The inverse-variance weighted (IVW) MR found no strong evidence for a causal effect of genetic liability to psoriasis on risk of COPD (OR = 1.01 per doubling of liability; 95% CI: 0.99–1.03; p = 3.89×10⁻¹). Heterogeneity was substantial (Q = 139, DF = 64; p = 2.04×10⁻⁷), suggesting possible pleiotropy. MR Egger provided evidence of a negative association (OR = 0.97; 95% CI: 0.95–0.99; p = 0.022) with an intercept significantly different from zero (p = 9.55×10⁻⁵), indicating directional pleiotropy. Both the weighted median (OR = 0.98; p = 0.055) and weighted mode (OR = 0.97; p = 0.018) gave slightly protective estimates.

Radial MR removed one outlier SNP rs17391694 which is not well characterised but has been implicated in both inflammation and cancer indicating pleiotropy. This adjustment reduced heterogeneity (Q = 119, DF = 63). After outlier removal, IVW results were unchanged (**OR = 1.01; 95% CI: 0.99–1.03; p = 0.495**). Directional filtering using Steiger's method did not materially change the results. Taken together, there is no consistent evidence that genetic liability to psoriasis increases COPD risk and observed associations. Removing the HLA region meant that a weak association became apparent.

**RA → COPD**

There was strong evidence that genetic liability to rheumatoid arthritis (RA) increases the risk of chronic obstructive pulmonary disease (COPD) in inverse-variance weighted (IVW) Mendelian randomization, with an odds ratio of 1.10 per doubling of RA liability (95% CI: 1.06–1.14; p = 3.4×10⁻⁸). However, this initial analysis showed substantial heterogeneity (Q = 113, DF = 59; p = 3.0×10⁻⁵), largely driven by variants in and around the HLA region on chromosome 6. Sensitivity analyses supported the association, with the weighted median (OR = 1.07; p = 2.0×10⁻⁶), weighted mode (OR = 1.06; p = 5.6×10⁻⁵), and MR GRIP (OR = 1.05; p = 3.7×10⁻³) showing consistent direction and significance. MR Egger returned a null estimate (OR = 1.03; p = 0.17), though the Egger intercept suggested weak directional pleiotropy (p = 0.025).

Radial MR removed two outliers in the RIN3; ELMO1 regions which have likely pleiotropic effects in osteoclast and lung function. This adjustment improved model fit (Q = 78, DF = 57; p = 0.036), slightly attenuating the IVW estimate to 1.09 (95% CI: 1.05–1.13; p = 8.6×10⁻⁹).

After Steiger filtering to retain directionally valid instruments, the final IVW estimate remained robust and significant (**OR = 1.06; 95% CI: 1.04–1.09; p = 8.7×10⁻⁹), supporting a causal effect of RA liability on COPD risk.** Instrument strength was high (mean F-statistic = 90.0). Removal of the HLA region strengthened this association slightly **Multivariable MR adjusting for asthma suggested modest attenuation in conventional analyses; however, this was not supported by weak-instrument-robust Qhet estimates.**

**PMR → COPD**

Initial inverse-variance weighted (IVW) Mendelian randomization suggested a modest causal effect of polymyalgia rheumatica (PMR) liability on chronic obstructive pulmonary disease (COPD), with an odds ratio of 1.07 (95% CI: 1.03–1.12; p = 0.0012). Heterogeneity was moderate (Q = 12.6, DF = 9; p = 0.18), and sensitivity analyses produced less precise estimates. MR Egger showed a directionally similar but non-significant effect (OR = 1.08; p = 0.054), while the weighted median (OR = 1.04; p = 0.15) and weighted mode (OR = 1.03; p = 0.49) were directionally consistent but not significant. MR GRIP also supported a small positive association (OR = 1.08; p = 0.028). The Egger intercept did not suggest directional pleiotropy (p = 0.78).

Radial MR analysis did not identify any influential outliers and yielded the same IVW estimate, reinforcing the robustness of the association.

**After Steiger filtering to retain only directionally valid instruments, the final IVW estimate remained significant (OR = 1.05; 95% CI: 1.02–1.08; p = 1.2×10⁻³), supporting a weak but consistent effect of PMR liability on COPD risk.** Instrument strength was high (mean F-statistic ≈ 131), minimizing concerns about weak instrument bias.

Testing for effect without HLA instruments was not possible due to the scarcity of valid instruments

Multivariable MR adjusting for asthma attenuated the association in conventional analyses; however, this was not supported by weak-instrument-robust Qhet estimates.

**OA → COPD**

The initial IVW analysis indicated a strong association (OR = 1.53; 95% CI: 1.43–1.63; p = 2.3×10⁻¹⁷) with extreme heterogeneity (Q = 337, DF = 128). Radial MR removed 5 outliers, improving heterogeneity (Q = 245, DF = 123), and Steiger filtering led to a refined estimate **(OR = 1.18; 95% CI: 1.13–1.24; p = 2.5×10⁻¹¹).** Other methods (median, mode, GRIP, Egger) were directionally consistent and mostly significant. No indication of pleiotropy was detected. Instrument strength was strong (F = 39.1). BMI explained a large part of this association in conventional multivariable MR analyses; however, this attenuation was not supported by weak-instrument-robust Qhet estimates.

**Psoriasis → Asthma**
The initial IVW analysis showed no association between genetic liability to psoriasis and asthma (OR = 1.00; 95% CI: 0.96–1.04; p = 9.02×10⁻¹), with extreme heterogeneity (Q = 451.0, DF = 64; p = 1.58×10⁻⁵⁹).
Radial MR removed 11 outliers in inflammatory regions such as TRIM65 and PTPN2 and slightly increased the point estimate (OR = 1.03; 95% CI: 1.00–1.05; p = 3.44×10⁻²), with reduced heterogeneity.
The final **Steiger-filtered IVW found a weak association (OR = 1.02; 95% CI: 1.00–1.04; p = 3.43×10⁻²).**
Weighted median (OR = 1.04; p = 4.66×10⁻³) and weighted mode (OR = 1.05; p = 3.70×10⁻²) estimates were directionally consistent.
MR Egger was not significant (OR = 0.99; p = 0.617), and the Egger intercept suggested mild directional pleiotropy (p = 0.014).

Excluding the HLA from the analysis increased the effect size psoriasis→asthma NO HLA 1.05 (95% CI: 1.03–1.08; p=7.00E-06)

**Rheumatoid Arthritis → Asthma**
The original IVW analysis suggested a strong association between genetic liability to RA and asthma (OR = 1.12; 95% CI: 1.07–1.17; p = 3.66×10⁻⁶), with substantial heterogeneity (Q = 289.3, DF = 57; p = 8.43×10⁻³³).
Sensitivity analyses supported this relationship: weighted median (OR = 1.09; p = 4.11×10⁻⁵), weighted mode (OR = 1.07; p = 2.16×10⁻²), MR GRIP (OR = 1.10; p = 1.18×10⁻²), and MR Egger (OR = 1.09; p = 7.73×10⁻²) were directionally consistent. The Egger intercept showed no significant pleiotropy (p = 0.66).
Radial MR removed outliers, strengthening the IVW estimate (OR = 1.12; 95% CI: 1.08–1.15; p = 2.54×10⁻¹¹) and reducing heterogeneity.
The final **Steiger-filtered IVW result** confirmed a robust and consistent **association (OR = 1.07; 95% CI: 1.05–1.10; p = 5.54×10⁻¹⁰**), supported by weighted median (OR = 1.09; p = 9.27×10⁻⁵), MR GRIP (OR = 1.10; p = 5.17×10⁻⁴), and MR Egger (OR = 1.09; p = 1.55×10⁻²).
These findings suggest a likely causal link from RA to asthma exclusion of the HLA regions showed an increase in the relationship RA→asthma 1.07 (95% CI: 1.04–1.10; p=1.86E-06)

**Polymyalgia Rheumatica (PMR) → Asthma**
The initial IVW analysis indicated a weak, non-significant association (OR = 1.04; 95% CI: 0.99–1.10; p = 0.126), with moderate heterogeneity (Q = 9.70, DF = 7; p = 0.21), though the analysis was limited by a small number of variants (n = 8).
Sensitivity methods—including weighted median (OR = 1.04; p = 0.201), MR GRIP (OR = 1.04; p = 0.318), and MR Egger (OR = 1.05; p = 0.344)—provided no evidence of causality, and the MR Egger intercept indicated no directional pleiotropy (p = 0.82).
Radial MR yielded identical estimates to the original analysis, and did not suggest outlier-driven bias.
The final Steiger-filtered IVW result remained non-**significant (OR = 1.02; 95% CI: 0.98–1.07; p = 0.287),** with similar null estimates from MR Egger (OR = 1.03; p = 0.615) and weighted median (OR = 1.03; p = 0.381).
Overall, there is **no evidence** for a causal effect of PMR on asthma, and the limited number and strength of instruments substantially reduce power to detect any modest association. It is not possible to test the association between the too and exclude the HLA regions

**Osteoarthritis (OA) → Asthma**
There was strong evidence for a causal effect of OA on asthma in the initial IVW analysis (OR = 1.26; 95% CI: 1.17–1.35; p = 8.63×10⁻¹¹), though heterogeneity was high (Q = 330.4, DF = 128; p = 7.80×10⁻²⁰). Sensitivity analyses provided consistent results: weighted median (OR = 1.19; p = 1.11×10⁻⁵), MR GRIP (OR = 1.27; p = 6.97×10⁻³), and MR Egger (OR = 1.28; p = 0.078). There was no evidence of directional pleiotropy (MR Egger intercept p = 0.898).
Radial MR excluded outliers and slightly attenuated the IVW estimate (OR = 1.24; 95% CI: 1.16–1.32; p = 6.00×10⁻¹⁰), with improved heterogeneity (Q = 297.6, DF = 126).
The **final Steiger-filtered IVW result confirmed a robust causal effect** (OR = 1.14; 95% CI: 1.09–1.19; p = 2.57×10⁻⁸), with consistent support across weighted median (OR = 1.18; p = 2.18×10⁻⁵), MR GRIP (OR = 1.29; p = 1.51×10⁻³), and MR Egger (OR = 1.36; p = 0.016). Instruments remained strong and pleiotropy minimal.
Removing the HLA regions from the MR did not change the results OA→asthma 1.14 (95% CI: 1.08–1.19; p=4.48E-08).

**Asthma to MSK conditions**

**Asthma → Psoriasis**
There was **no evidence of a causal effect** in the initial IVW analysis (OR = 1.04; 95% CI: 0.95–1.14; p = 0.368), and MR Egger (OR = 1.05; p = 0.676), weighted median (OR = 1.08; p = 0.079), and MR GRIP (OR = 1.03; p = 0.715) were similarly non-significant. No pleiotropy was detected (Egger intercept p = 0.963).
However, **radial MR** excluding outliers revealed a significant IVW estimate (OR = 1.13; 95% CI: 1.05–1.21; p = 4.59×10⁻⁴), supported by weighted median (OR = 1.08; p = 0.049), MR GRIP (OR = 1.15; p = 0.010), and MR Egger (OR = 1.17; p = 0.047).
The **final Steiger-filtered IVW analysis confirmed a modest causal effect** (OR = 1.05; 95% CI: 1.01–1.10; p = 0.014), supported by MR GRIP (OR = 1.13; p = 0.018) and MR Egger (OR = 1.17; p = 0.041). Weighted median and mode estimates were attenuated. The small association may largely be driven through the HLA region as the association reduced without these regions asthma→psoriasis 1.03 (95% CI: 0.99–1.08; p=1.03E-01).

**Asthma → Rheumatoid Arthritis (RA)**
Initial IVW analysis provided **strong evidence of a causal effect** (OR = 1.35; 95% CI: 1.22–1.49; p = 6.46×10⁻⁹), supported by MR Egger (OR = 1.76; p = 6.56×10⁻⁶), weighted median (OR = 1.18; p = 5.42×10⁻⁵), and MR GRIP (OR = 1.50; p = 1.65×10⁻⁶). However, potential pleiotropy was detected (Egger intercept p = 0.017).

Following **Radial MR outlier removal,** the IVW effect remained consistent (OR = 1.30; 95% CI: 1.22–1.39; p = 2.54×10⁻¹⁴), and was further supported by weighted median (OR = 1.35; p = 1.10×10⁻¹⁴), MR Egger (OR = 1.31; p = 6.67×10⁻³), and weighted mode (OR = 1.53; p = 6.13×10⁻⁶). The Egger intercept after outlier removal was null (p = 0.94), suggesting pleiotropy was mitigated.

SNPs removed included varaints in *IL33*, *IL1RL1*, *TSLP*, *IL6R*, *HLA-DQA1.*

In the **final Steiger-filtered analysis**, a robust IVW estimate persisted (**OR = 1.19; 95% CI: 1.13–1.24; p = 1.02×10⁻¹²),** with strong concordance from weighted median (OR = 1.35; p = 2.88×10⁻¹³), MR Egger (OR = 1.37; p = 1.28×10⁻³), and MR GRIP (OR = 1.32; p = 1.48×10⁻⁵).

This result was slightly reduced by removal of the HLA regions from the analysis asthma→RA 1.12 (95% CI: 1.07–1.18; p=1.98E-06).

**Asthma → Polymyalgia Rheumatica (PMR)**
Initial IVW analysis suggested a modest positive association (OR = 1.16; 95% CI: 1.05–1.29; p = 4.46×10⁻³), with weak support from MR GRIP (OR = 1.18; p = 4.79×10⁻²). However, the weighted median (p = 0.081) and MR Egger (p = 0.078) were non-significant, and the Egger intercept showed no evidence of directional pleiotropy (p = 0.537).

Following **Radial MR outlier removal,** the IVW estimate attenuated to non-significance (OR = 1.10; 95% CI: 0.99–1.21; p = 0.072), with similar null findings from other methods including MR GRIP (OR = 0.99; p = 0.897).

In the **final Steiger-filtered analysis,** no evidence of a causal effect remained (**IVW OR = 1.01; 95% CI: 0.95–1.08; p = 0.734),** and all sensitivity methods—including weighted median, mode, MR Egger, and MR GRIP—were non-significant.

These results **do not support a robust causal effect of asthma on PMR**, and any initial signal appears sensitive to outlier and directional filtering, suggesting possible confounding or pleiotropy rather than a true causal link.

**Asthma → Osteoarthritis (OA)**
The initial IVW analysis indicated a modest association between asthma and OA (OR = 1.04; 95% CI: 1.02–1.07; p = 1.00×10⁻³), but with notable heterogeneity (Q = 406.1, p = 2.41×10⁻²³). MR Egger yielded a null and directionally opposite estimate (OR = 0.97; p = 0.38), and the MR Egger intercept suggested potential directional pleiotropy (p = 0.014).

After **Radial MR outlier removal**, the IVW estimate remained significant (OR = 1.04; 95% CI: 1.02–1.06; p = 2.61×10⁻⁴), and the weighted mode became borderline significant (OR = 1.04; p = 0.047). However, both MR Egger and MR GRIP continued to return non-significant results, and pleiotropy was again suggested by the Egger intercept (p = 0.020).

In the **Steiger-filtered final analysis**, the IVW association persisted **(OR = 1.03; 95% CI: 1.02–1.05; p = 4.50×10⁻⁵).** The weighted median (OR = 1.05; p = 4.91×10⁻⁴) and weighted mode (OR = 1.04; p = 0.048) also supported this association. However, MR Egger remained null (p = 0.695), and the Egger intercept continued to indicate pleiotropy (p = 0.030).

Altogether, **a small but statistically significant association** was detected between asthma and OA, robust to filtering and supported by median- and mode-based methods. However, the presence of **directional pleiotropy and inconsistency across MR models**—particularly the null Egger estimate—suggests this association may be **partially confounded or pleiotropy-driven**. Multivariable MR analyses indicated partial attenuation after adjustment for IL6, which was supported by weak-instrument-robust Qhet estimates, suggesting a role for systemic inflammatory pathways in this relationship.

# Observational Note on Coding

CPRD AURUM data captures demographic, diagnostic, drug, lab and referral information from patients attending 1,491 UK-based GPs, distributed across England. The data is regularly collected and updated with de-identified data made available to researchers upon acceptance of a study protocol. The extract contains several files including Observation, Drug Issue, Patient, Staff, Practice, Referral and Consultation. This dataset includes data on over 40 million patients, of which 13,300,067, 19.8% of the English population are currently alive and enrolled with a practice. Patients included in CPRD AURUM are broadly representative of the English population. Diagnosis in CPRD are recorded using Read Codes v2, EMIS codes and SNOMED CT codes. In this study, we included N=11,046,182 patients alive, registered and over the age of 40 on 01/01/2020. Patients were considered to have a disease with at least one observation matching the code list at index date.

SIDIAP captures demographic, diagnostic, drug, lab and referral information from patients attending 328 primary-care centres that cover 75% of the population living in Catalonia, Spain. The database is updated every 6 months and is structured in data domains, each containing the person’s pseudo-anonymised identifier. Data is accessible to all researchers following approval of a research protocol. The extract contains information on Socio-demographics, Health conditions, Medications and vaccines, Laboratory tests, Clinical practice and lifestyle information, and Sexual and reproductive health. The database has information on 8,036,948 people, of whom 5,801,280 (72.2%) were still active as of 30 June 2021. SIDIAP is representative of the population of Catalonia in terms of age, sex and geographic distribution. SIDIAP the electronic codes are recorded using the International Classification of Disease, version 10 (ICD-10-CM/PCS).^6^ In this study, we included N= 2,625,989 patients alive, registered and over the age of 40 on 01/01/2020 were analysed. Patients were considered to have a disease with at least one observation matching the code list at index date.

Mapping between different code vocabularies was achieved using concept codes [7].

# Strobe MR

*Please find attached as COPD_strobe_MR.pdf*

# Observational RECORD Checklist

*Please find attached as RECORD observationsl_checklist_COPD_plos.pdf*

# Supplementary Table Captions

*All tables provided in the accompanying zipped Excel file)*

**Supplementary Table 1.**

**Musculoskeletal (MSK) and respiratory condition pairs analysed in genetic correlation and observed co-occurrence.**
Includes condition definitions, sample sizes from UK Biobank, FinnGen, and relevant consortia, and previously published genetic correlations from Murrin et al. [8].

**Supplementary Table 2.**

**Category definitions for non-disease covariates used in observational analyses**
Includes biometric, lifestyle, and demographic variables, with categorisation schemes used in CPRD and SIDIAP analyses. Comments on whether they have also been instrumented in genetic analyses where summary statistics were available.

**Supplementary Table 3.**

**Observational and mediation datasets (CPRD): baseline characteristics of exposed groups (Disease A) and matched controls.**
Includes age, sex, deprivation, comorbidities, lifestyle factors, and biomarker/biometric variables used in overlap weighting and mediator eligibility assessment.

**Supplementary Table 4.**

**Observational datasets (SIDIAP): baseline characteristics of exposed groups (Disease A) and matched controls.**
Includes age, sex, deprivation, comorbidities, lifestyle factors, and biomarker/biometric variables for replication analyses.

**Supplementary Table 5.**

**Prognostic consequences dataset (CPRD): baseline characteristics of groups with Disease A, Disease B, and multimorbidity (A&B), and matched controls.**
Includes demographic, lifestyle, comorbidity, and biomarker variables used for mortality and hospitalisation models.

**Supplementary Table 6.**

**Two-sample Mendelian randomisation (MR) results for diseases causing each risk factor.**
Preliminary MR screen used to prioritise candidate mediators for observational mediation analysis.

**Supplementary Table 7.**

**Z-test comparing unadjusted and adjusted observational effect estimates to identify candidate mediators.**
Reports change in effect size, and significance for each risk factor across disease pairs.

**Supplementary Table 8.**

**Full Mendelian randomisation results for all respiratory–MSK disease pairs.**
Includes IVW, weighted median, weighted mode, MR-Egger, Radial MR outlier-corrected results, Steiger filtering, and HLA-exclusion sensitivity analyses.

**Supplementary Table 9.**

**Summary of instrument filtering in Mendelian randomisation analyses.**
Includes SNP counts before and after Steiger filtering, Radial MR outlier removal

**Supplementary Table 10.**

**Summary of genetic instruments and effect sizes used across all MR analyses.**
Includes effect allele, beta/SE or OR/SE, allele frequency

**Supplementary Table 11.**

**MVMR sensitivity analyses**
MVMR for select potential confounders odds ratios are presented on the exp(b) scale. these analyses were run prior to any instrument filtering (raw) and after attempted removal of pleiotropy. Q_het is a calculation that was more robust against weak instruments therefore this was the deciding factor in whether a risk factor attenuated a pairwise association.

**Supplementary Table 12**

**Temporal diagnostic sequences and estimated windows for intervention for each respiratory–MSK disease pair.**
Mean and median ages at diagnosis for Diseases A and B; proportions of A→B and B→A sequences; mean and median times between diagnoses; SD and IQR.

**Supplementary Table 13.**

**Bidirectional MR results for risk factors influencing respiratory or MSK conditions.**
Causal estimates for BMI, waist-hip ratio, lipid fractions, education, smoking traits, alcohol consumption, and physical activity.

**Supplementary Table 14.**

**Time-to-event mediation analysis results (CPRD).**
Direct, indirect, and total effects; proportion mediated; confidence intervals; and FDR-adjusted p values for mediators that met MR and Z-test selection criteria.

**Supplementary Table 15.**

**Colocalisation results across shared genomic regions for respiratory–MSK disease pairs.**
number of SNPs per region, probability of a shared causal variant (PPH4), and effect direction across traits.

**Supplementary Table 16.**

**Proteomic follow-up of colocalised variants and druggability assessment.**
List of cis- and trans-pQTLs for implicated proteins, evidence from Olink proteomics, druggability annotation (DrugBank, DGIdb), and corresponding drug targets.

# References

1.	Yengo L, Sidorenko J, Kemper KE, Zheng Z, Wood AR, Weedon MN, et al. Meta-analysis of genome-wide association studies for height and body mass index in ∼700000 individuals of European ancestry. Hum Mol Genet. 2018;27(20):3641-9. doi: 10.1093/hmg/ddy271. PubMed PMID: 30124842; PubMed Central PMCID: PMCPMC6488973.

2.	Pulit SL, Stoneman C, Morris AP, Wood AR, Glastonbury CA, Tyrrell J, et al. Meta-analysis of genome-wide association studies for body fat distribution in 694 649 individuals of European ancestry. Hum Mol Genet. 2019;28(1):166-74. doi: 10.1093/hmg/ddy327. PubMed PMID: 30239722; PubMed Central PMCID: PMCPMC6298238.

3.	Graham SE, Clarke SL, Wu K-HH, Kanoni S, Zajac GJM, Ramdas S, et al. The power of genetic diversity in genome-wide association studies of lipids. Nature. 2021;600(7890):675-9. doi: 10.1038/s41586-021-04064-3.

4.	Okbay A, Wu Y, Wang N, Jayashankar H, Bennett M, Nehzati SM, et al. Polygenic prediction of educational attainment within and between families from genome-wide association analyses in 3 million individuals. Nature Genetics. 2022;54(4):437-49. doi: 10.1038/s41588-022-01016-z.

5.	Liu M, Jiang Y, Wedow R, Li Y, Brazel DM, Chen F, et al. Association studies of up to 1.2 million individuals yield new insights into the genetic etiology of tobacco and alcohol use. Nat Genet. 2019;51(2):237-44. Epub 20190114. doi: 10.1038/s41588-018-0307-5. PubMed PMID: 30643251; PubMed Central PMCID: PMCPMC6358542.

6.	Wang Z, Emmerich A, Pillon NJ, Moore T, Hemerich D, Cornelis MC, et al. Genome-wide association analyses of physical activity and sedentary behavior provide insights into underlying mechanisms and roles in disease prevention. Nat Genet. 2022;54(9):1332-44. Epub 20220907. doi: 10.1038/s41588-022-01165-1. PubMed PMID: 36071172; PubMed Central PMCID: PMCPMC9470530.

7.	Reich C, Ostropolets A, Ryan P, Rijnbeek P, Schuemie M, Davydov A, et al. OHDSI Standardized Vocabularies—a large-scale centralized reference ontology for international data harmonization. Journal of the American Medical Informatics Association. 2024;31(3):583-90. doi: 10.1093/jamia/ocad247.
